# Metformin plus lifestyle interventions versus lifestyle interventions alone for the delay or prevention of type 2 diabetes in individuals with prediabetes: a meta-analysis of randomized controlled trials

**DOI:** 10.1186/s13098-024-01504-8

**Published:** 2024-11-14

**Authors:** Basma Ehab Amer, Mahmoud Shaaban Abdelgalil, Abdullah Ashraf Hamad, Kerollos Abdelsayed, Ahmed Elaraby, Ahmed Mohamed Abozaid, Mohamed Abd-ElGawad

**Affiliations:** 1 Faculty of Medicine, Benha University, Benha, Egypt; 2 Faculty of Medicine, Ain-shams University, Cairo, Egypt; 3 Faculty of Medicine, Menoufia University, Menoufia, Egypt; 4 Clinical Research Department, Magdi Yacoub Foundation, Aswan Heart Center, Aswan, Egypt; 5 Faculty of Medicine, Al-Azhar University, Cairo, Egypt; 6 Faculty of Medicine, Tanta University, Tanta, Egypt; 7 Faculty of Medicine, Fayoum University, Fayoum, Egypt

**Keywords:** Prediabetes, Type 2 diabetes, HbA1c, FPG, Metformin, Lifestyle

## Abstract

**Objectives:**

We conducted this meta-analysis of randomized controlled trials (RCTs) to compare the efficacy of adding metformin to lifestyle interventions versus lifestyle interventions alone in individuals with prediabetes.

**Materials and methods:**

We searched four databases from inception until March 20, 2024. Our primary outcomes included the incidence of type 2 diabetes, hemoglobin A1c (HbA1c), and fasting plasma glucose (FPG). Secondary outcomes included blood pressure, plasma lipids, and weight measurements. Dichotomous outcomes were pooled as the risk ratio (RR) and its 95% confidence interval (CI), while continuous outcomes were pooled as the standardized mean difference (SMD) and its 95% CI in the random effect model. All statistical analyses were conducted using the “meta” package of RStudio software.

**Results:**

We included 12 RCTs, comprising 2720 patients. Adding metformin to lifestyle interventions significantly reduced HbA1c levels (SMD = -0.10, 95% CI [-0.19, -0.01], *P* = 0.03) and the incidence of type 2 diabetes (RR = 0.85, 95% CI [0.75, 0.97], *P* = 0.01). Interestingly, adding metformin to lifestyle interventions was comparable to lifestyle interventions alone in terms of FPG at both 3 and 6 months; however, it significantly reduced FPG at 12 months (SMD = -0.34, 95% CI [-0.59, -0.08], *P* = 0.01). There were no significant differences between the two groups in terms of all secondary outcomes.

**Conclusions:**

Our findings suggest that adding metformin to lifestyle interventions may improve glycemic control in individuals with prediabetes and reduce their risk of progression to diabetes, compared to lifestyle interventions alone. A longer duration of this combined approach may be required to observe the desired effects.

**Supplementary Information:**

The online version contains supplementary material available at 10.1186/s13098-024-01504-8.

## Introduction

About 537 million adults are living with diabetes. Of them, more than 90% are diagnosed with type 2 diabetes. Given the continuous rise in the global incidence and prevalence of diabetes, it is considered a global pandemic ranking among the leading causes of premature death. It is predicted that 783 million individuals will be affected with diabetes by 2045 [1].

Prediabetes, which is a major risk factor for the development of type 2 diabetes, is an intermediate stage of hyperglycemia in which blood glucose levels are below the diagnostic threshold for type 2 diabetes; however, they are too high to be normal. Individuals with prediabetes have either impaired glucose tolerance, impaired fasting glucose, or both [2]. The American Diabetes Association (ADA) defines impaired fasting glucose as a fasting glucose level of 100 to 125 mg/dL, while impaired glucose tolerance is defined as a 2-hour plasma glucose level after a 75-g oral glucose challenge (2hPG) of 140 to 199 mg/dL [2, 3]. Prediabetes can also be diagnosed with an HbA1c level of 5.7–6.4% [4]. The major risk factors for prediabetes include obesity, physical inactivity, older age, and genetic predisposition [5]. Given the increasing prevalence of obesity across all age groups [6], the International Diabetes Federation expects that by 2045, one billion individuals will be affected with prediabetes [7].

Individuals with prediabetes have a higher risk of developing type 2 diabetes, cardiovascular complications, peripheral neuropathy, and accelerated frailty compared to those with normal glucose regulation [8–11]. Hence, we can reduce these complications by adopting effective strategies to decrease the transition from prediabetes to diabetes [12, 13]. Currently, lifestyle interventions, such as calorie restriction, nutrition visits, and exercise are the mainstay of diabetes prevention. However, adherence to these lifestyle interventions is challenging [5, 14].

Therefore, multiple randomized controlled trials (RCTs) have investigated the efficacy of pharmacological agents, such as metformin, glucagon-like peptide 1 (GLP-1) analogs, and thiazolidinediones for delaying or even preventing the progression from prediabetes to diabetes [15–19]. Although metformin was superior to placebo in reducing the progression from prediabetes to diabetes, its long-term efficacy for preventing diabetes was generally lower than that of lifestyle interventions [13, 17, 20]. Most diabetes prevention trials have found that the effects of lifestyle interventions on diabetes prevention persisted after discontinuation of these interventions; however, metformin lost its effect when discontinued [21, 22].

To date, the efficacy of adding lifestyle interventions to metformin versus lifestyle interventions alone in individuals with prediabetes is still inconclusive. Previous meta-analyses revealed that adding metformin to lifestyle interventions was comparable to lifestyle interventions alone [20, 23]. Nevertheless, these meta-analyses had some problems. First, they included a small number of RCTs. Second, they did not specifically compare the effect of adding metformin to lifestyle interventions versus lifestyle interventions alone [20, 23]. Furthermore, published RCTs, which were not included in the previous meta-analyses, showed conflicting results [18, 24–35]. Moreover, in multiple subgroup analyses of the Diabetes Prevention Program, metformin showed a protective impact on lipid control, inflammation, metabolic syndrome, and coronary artery calcium; suggesting benefits for metformin in individuals with prediabetes that extend beyond diabetes prevention [36–39]. Finally, although the ADA guidelines recommended considering metformin for certain individuals with prediabetes [40], the adherence of physicians to these recommendations remains unclear, particularly with contradicting views in the literature on the true benefit of the medication [41, 42].

Therefore, we conducted this systematic review and meta-analysis to update the evidence from all published RCTs, comparing the clinical and biochemical effectiveness of adding metformin to lifestyle interventions versus lifestyle interventions alone in individuals with prediabetes.

## Methods

We followed the preferred reporting items for systematic reviews and meta-analysis (PRISMA) statement guidelines when performing this systematic review and meta-analysis [43]. In addition, our study was carried out per the Cochrane Handbook of Systematic Reviews and Meta-analysis of Interventions [44]. We registered our study protocol on the PROSPERO database (CRD42023458096).

### Literature search

On August 1, 2023, we performed a comprehensive literature search on four electronic databases (PubMed, Scopus, Web of Science, and Cochrane Central) using the following keywords: lifestyle, metformin, prediabetes, glucose intolerance, and impaired fasting glucose. In addition, we performed manual citation analysis for all the references of the included studies and relevant systematic reviews and meta-analyses. On March 20, 2024, we updated our search again for other potential publications. We provide the detailed search strategy and results for each database in Supplementary Table 1.

### Eligibility criteria

We considered all RCTs comparing lifestyle interventions plus metformin versus lifestyle interventions alone or lifestyle interventions plus placebo in individuals with prediabetes either with impaired glucose tolerance, impaired fasting glucose, or both. Our population was not restricted to a specific age group. Therefore, RCTs including adults or adolescents were included. Lifestyle interventions were defined as diet or exercise interventions offering more than the provision of general information or advice, which are considered “standard care”. However, we were interested in more intensive care such as goal setting and individually tailored information to ensure the implementation of these interventions in the study population. Included studies had to assess at least one of our outcome measurements. We excluded studies comparing adding metformin to lifestyle interventions versus metformin alone. Similarly, studies comparing lifestyle interventions versus metformin were also excluded. RCTs including individuals with concomitant use of other antidiabetic drugs were excluded. Finally, animal studies, conference abstracts, and studies that were not in English were excluded.

### Primary and secondary outcomes

Our primary outcomes included the incidence of type 2 diabetes and glycemic control measurements, such as HbA1c and FPG levels, which are important indicators of diabetes risk. Secondary outcomes included clinical and biochemical parameters, which provide insights about the overall health status and risk factors associated with diabetes, such as blood pressure, plasma lipids, insulin resistance, and weight measurements (Table 1).

**Table 1:** Secondary outcomes

Secondary outcome	Measurements
• Plasma lipids	• Serum triglycerides (mg/dl)
	• Serum total cholesterol (mg/dl)
	• Serum HDL (mg/dl)
	• Serum LDL (mg/dl)
• Blood pressure	• Diastolic blood pressure (mmHg)
	• Systolic blood pressure (mmHg)
• Body weight	• Weight (Kg)
	• BMI (kg/m2)
	• Waist circumference (cm)
• Insulin resistance	• HOMA-IR

### Screening of the literature search results

The literature search results were screened in a two-step process. Initially, the titles and abstracts of all articles were assessed for eligibility. Subsequently, full-text screening was conducted for the studies that met our eligibility criteria.

### Data extraction

Data from the included studies was extracted in a standardized data extraction sheet, which was formulated by one author and agreed upon by all other authors. The extracted data encompassed four main categories: (1) Summary of the included studies, such as the study ID, country, total sample size, follow-up duration, and key findings, (2) Baseline characteristics of the study population, such as the mean age, FPG, and HbA1c levels, (3) Risk of bias domains, and (4) Primary and secondary outcomes.

### Synthesis of results

For outcomes that involved dichotomous data, the frequency of events and the total number of patients in each group were combined to calculate the relative risk (RR) and its 95% confidence interval (CI). For outcomes that involved continuous data, we used the standardized mean difference (SMD) and its 95% confidence interval as our effect estimate. We used the random effect model for all outcomes to account for the suspected heterogeneity among the included studies. In cases where studies reported data at multiple time points, the last endpoint was considered for the primary analysis. We also extracted the data at different time points and conducted subgroup analyses to investigate the efficacy change over time. Moreover, we conducted a subgroup analysis based on the age group (adults versus adolescents) and the geographical distribution of the included RCTs (Asia versus America). At least two RCTs are required for each subgroup. If only one of the adolescents studies was pooled with adult studies, subgroup analysis was not applicable. Therefore, we performed sensitivity analysis omitting this study and reported our findings both beforeand after omitting it. All statistical analyses were conducted using the R (v.4.3.0) programming language and the “meta” package of RStudio software for Windows [45].

### Assessment of heterogeneity

The presence of statistical heterogeneity among the included RCTs was assessed using the Cochrane Q test, which calculates the chi-square statistic. A p-value less than 0.1 for the Chi-square test was indicative of significant heterogeneity.

### Sensitivity analysis

We performed sensitivity analyses using the leave-one-out method to evaluate the robustness of our evidence. For each outcome included in the meta-analysis, we conducted sensitivity analysis in various scenarios by excluding one study at a time, to ensure that the overall effect estimate was not heavily influenced by any single study [46].

### Quality assessment

Two authors independently evaluated the quality of the included clinical trials using the Cochrane Risk of Bias 2 tool for RCTs, which involves five domains: randomization process (selection bias), deviation from intended interventions (performance bias), outcome measurement (detection bias), missing outcome data (attrition bias), selection of reported results (reporting bias), and other potential sources of bias [47]. The authors’ assessment decisions were categorized as ‘Low risk of bias’, ‘High risk of bias’, or ‘Some concerns’. Any discrepancies between the two authors were resolved through discussion with a third author.

### Publication bias

According to Egger et al., publication bias assessment is reliable only for at least 10 pooled studies [48]. Although our meta-analysis included 12 RCTs, we could not assess the risk of publication bias due to the insufficient number of pooled RCTs in each outcome.

## Results

### Literature search results

**Fig. 1:**
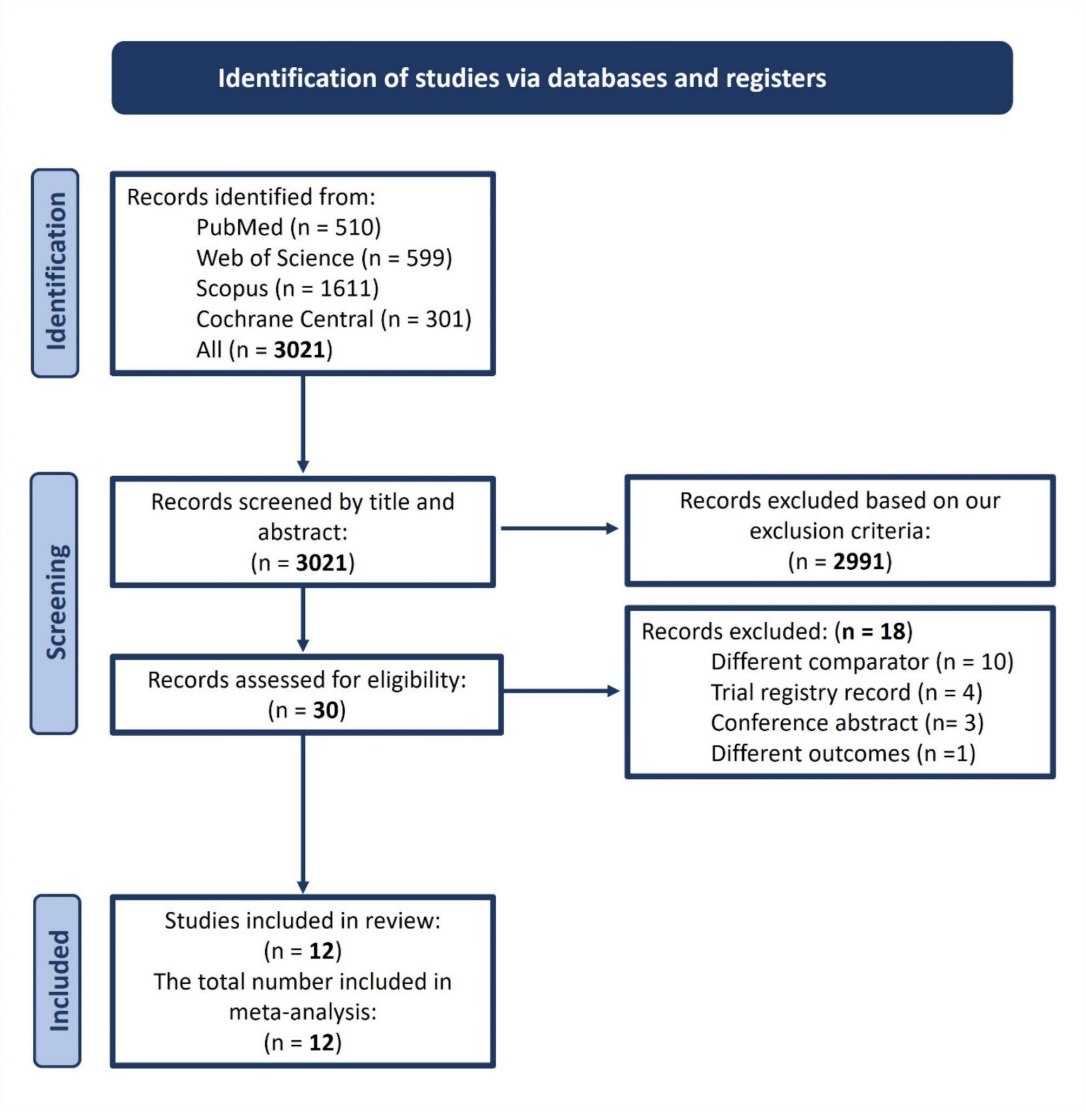
The PRISMA flow diagram of the study selection process

Our comprehensive search identified a total of 3021 records. The titles and abstracts of all these records were screened; however, only 30 articles seemed eligible for full-text screening. Of them, 12 RCTs were included in our systematic review and meta-analysis. No further RCTs were identified after performing citation analyses. We provide the PRISMA flow diagram of the study selection process in Fig. 1.

### Characteristics of individual studies

**Fig. 2:**
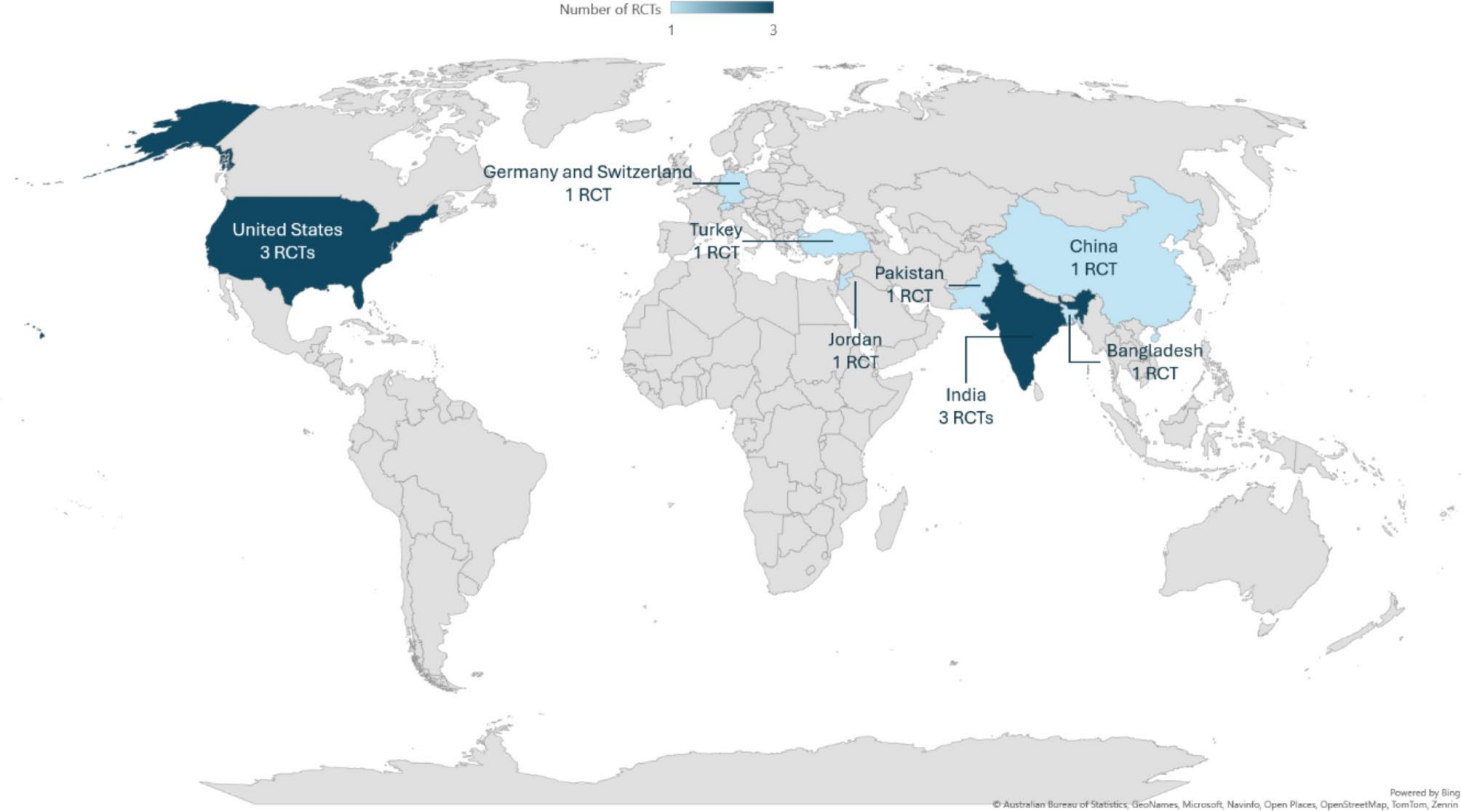
Geographical distribution of the randomized controlled trials included in our meta-analysis

Our meta-analysis included 12 RCTs, comprising 2720 individuals with prediabetes. The largest sample size was in Zhang et al., which included 1678 participants [18], while the smallest sample size was in Malin et al., which included 16 participants [31]. Three studies were conducted in India [8, 25, 30] and three were conducted in the United States [28, 31, 32]. Figure 2 shows the geographical distribution of all RCTs included in our meta-analysis [18, 24, 26, 27, 34, 35]. The follow-up duration ranged from three months in both Viskochil et al. [28] and Malin et al. [31] studies to 24 months in Zhang et al. [18]. We summarized all included studies and their patients’ baseline characteristics in Table 2 and Supplementary Table 2, respectively. In addition, Supplementary Table 3 summarizes the lifestyle interventions used by each of the included RCTs.

According to the ROB-2 tool, three RCTs showed a low risk of bias; six RCTs demonstrated some concerns regarding their risk of bias; and the remaining three RCTs showed a high risk of bias. A summary and graph for risk of bias assessment are shown in Supplementary Fig. S1a and S1b, respectively.

**Table 2:** Summary of the included studies

Study ID	Country	Total sample size	Follow-up duration (months)	Type of prediabetes (IGT, IFG, or both)	Key findings
Arslan et al. [27]	Turkey	54	12	Both	• Patients in the LSI with metformin group had significantly lower waist circumference, body mass index, and insulin resistance than those in the LSI only group. • There was no significant difference between the two groups in terms of lipid profile outcomes.
Barua et al. [26]	Bangladesh	100	12	Both	• The group with LSI plus metformin exhibited more significant changes in triglyceride levels and demonstrated more substantial improvements in BMI.
Basavareddy et al. [25]	India	104	12	Both	• There was no significant difference between patients in the LSI with metformin versus the LSI only groups in terms of abdominal circumference, total cholesterol, triglycerides, fasting blood sugar, and HbA1c reduction form baseline.
Bulatova et al. [35]	Jordan	53	6	NR	• Metformin combined with LSI significantly reduced BMI and HbA1c compared to LSI alone.
Hydrie et al. [34]	Pakistan	209	18	IGT	• LSI was highly effective in preventing individuals with impaired glucose tolerance from progressing to diabetes. • The addition of metformin to LSI was not found to provide an additional advantage.
Kulkarni et al. [8]	India	70	6	Both	• Both LSI with metformin and LSI alone reduced weight and FPG. However, reduced HbA1c was observed only in the LSI with metformin group.
Love-Osborne et al. [32]	USA	85	6	Both	• There was no significant difference between patients in the LSI plus metformin group versus those in the LSI plus placebo in terms of BMI reduction.
Malin et al. [31]	USA	16	3	Both	• There was no significant difference between patients in the exercise training with placebo and exercise training with metformin groups in terms of insulin sensitivity.
Ramachandran et al. [30]	India	262	36	IGT	• The combination of LSI and metformin did not provide any additional advantage in reducing diabetes incidence among Asian Indians with IGT.
Viskochil et al. [28]	USA	19	3	IGT	• Metformin plus LSI was comparable to LSI alone in terms of FPG reduction.
Wiegand et al. [24]	Germany and Switzerland	70	6	Both	• During the LSI phase, there was a significant deterioration in both BMI and insulin resistance. • In the following medication phase, improvements in insulin resistance and fasting insulin were observed in both the placebo and metformin groups, with no significant changes in BMI between the two groups.
Zhang et al. [18]	China	1678	24	Both	• Metformin plus LSI significantly reduced the risk of diabetes development compared to LSI alone.

IGT, impaired glucose tolerance; IFG, impaired fasting glucose; USA, United States of America; LSI, lifestyle interventions; BMI, body mass index; FPG, fasting plasma glucose; HbA1c, glycated hemoglobin; NR, not reported.

### Outcome measures

#### Incidence of type 2 diabetes

**Fig. 3:**
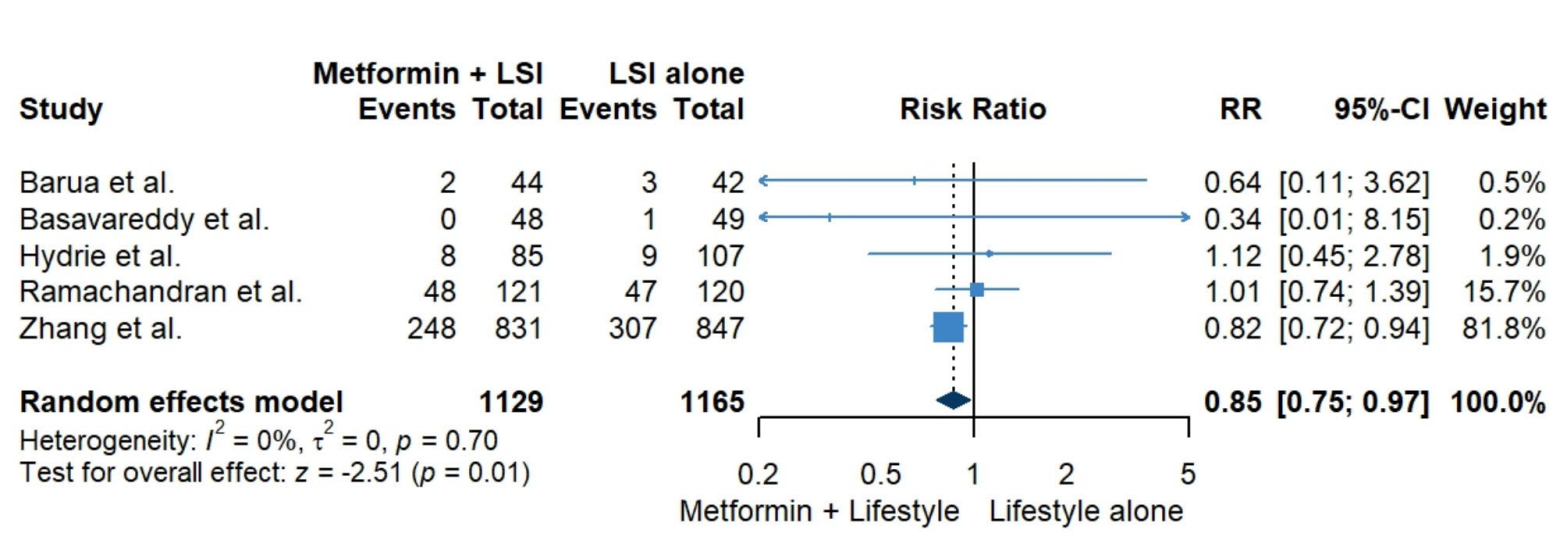
Pooled results for the incidence of type 2 diabetes

Our pooled analysis revealed a significant reduction in the incidence of type 2 diabetes when metformin was combined with lifestyle interventions compared to lifestyle interventions alone (RR = 0.85, 95% CI [0.75, 0.97], *P* = 0.01). The pooled studies were homogenous (I^2^ = 0%, *P* = 0.70) (Fig. 3). However, sensitivity analysis omitting Zhang et al. revealed no significant difference between the two groups (RR = 1, 95% CI [0.75, 1,34], I^2^ = 0%) (Table 3 and Supplementary Fig. S2).

#### Glycemic control measurements

##### HbA1c

**Fig. 4:**
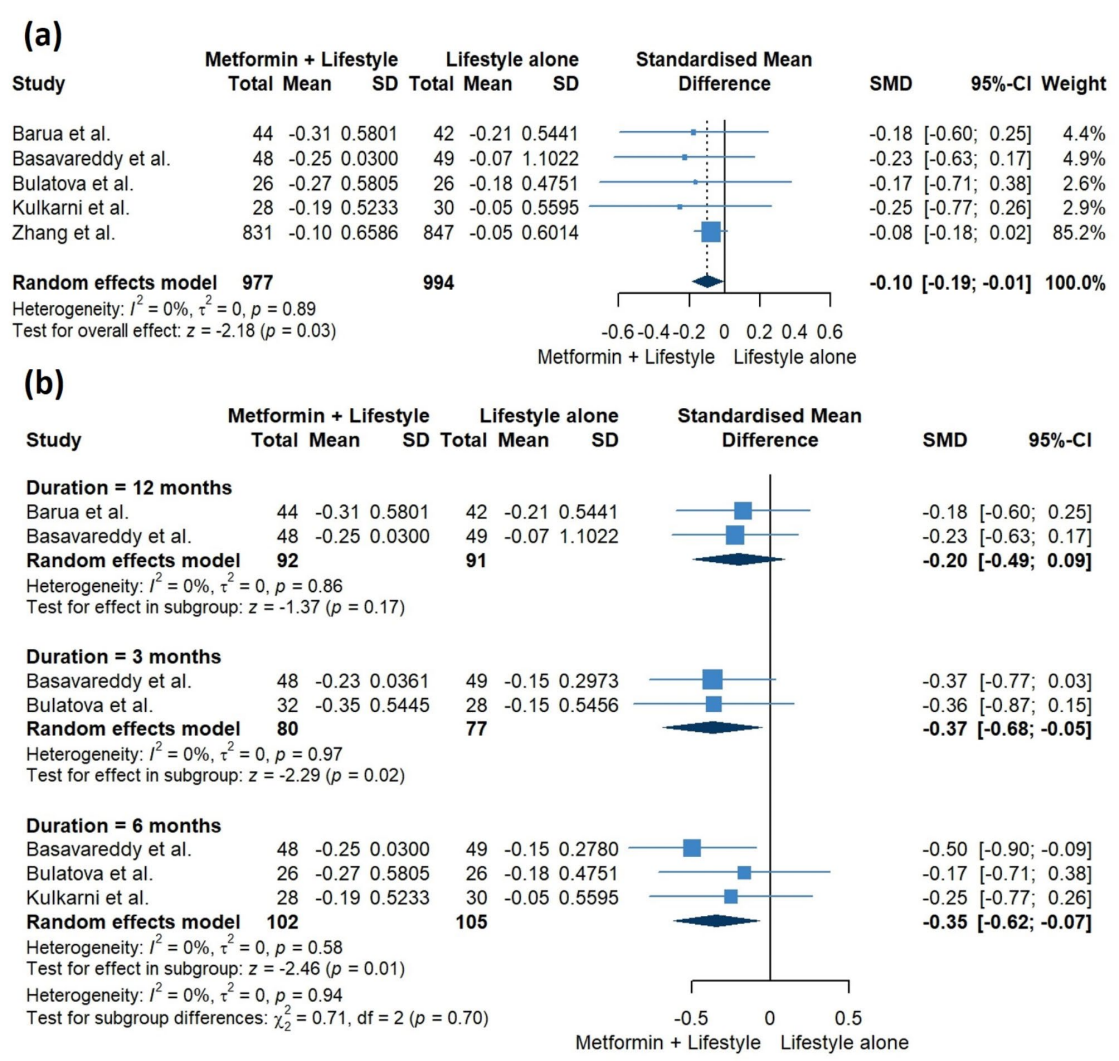
Pooled results for the change in HbA1c levels at **(a)** endpoints and **(b)** different time points

Our pooled analysis for the change in HbA1c levels at the endpoints of included studies showed that the combination of metformin and lifestyle interventions exhibited a significant reduction in HbA1c levels, compared to lifestyle interventions alone (SMD = -0.10, 95% CI [-0.19, -0.01], *P* = 0.03). The pooled studies were homogenous (I^2^ = 0%, *P* = 0.89) **(**Fig. 4a**)**. However, sensitivity analysis omitting Zhang et al. showed no significant difference between the two groups (RR = -0.21, 95% CI [-0.44, 0.02], I^2^ = 0%) (Table 3 and Supplementary Fig. S3).

Furthermore, our subgroup analysis based on different time points, revealed that the combined approach resulted in a significant decrease in HbA1c levels at 3 and 6 months (SMD = -0.37, 95% CI [-0.68, -0.05], *P* = 0.02; SMD = -0.35, 95% CI [-0.62, -0.07], *P* = 0.01, respectively). However, the pooled studies at 12 months indicated no significant difference between the two groups (SMD = -0.20, 95% CI [-0.49, 0.09], *P* = 0.17). The studies within each subgroup exhibited homogeneity (I^2^ = 0%, *P* = 0.97; I^2^ = 0%, *P* = 0.58; I^2^ = 0%, *P* = 0.86, respectively) (Fig. 4b).

##### Fasting blood glucose

**Fig. 5:**
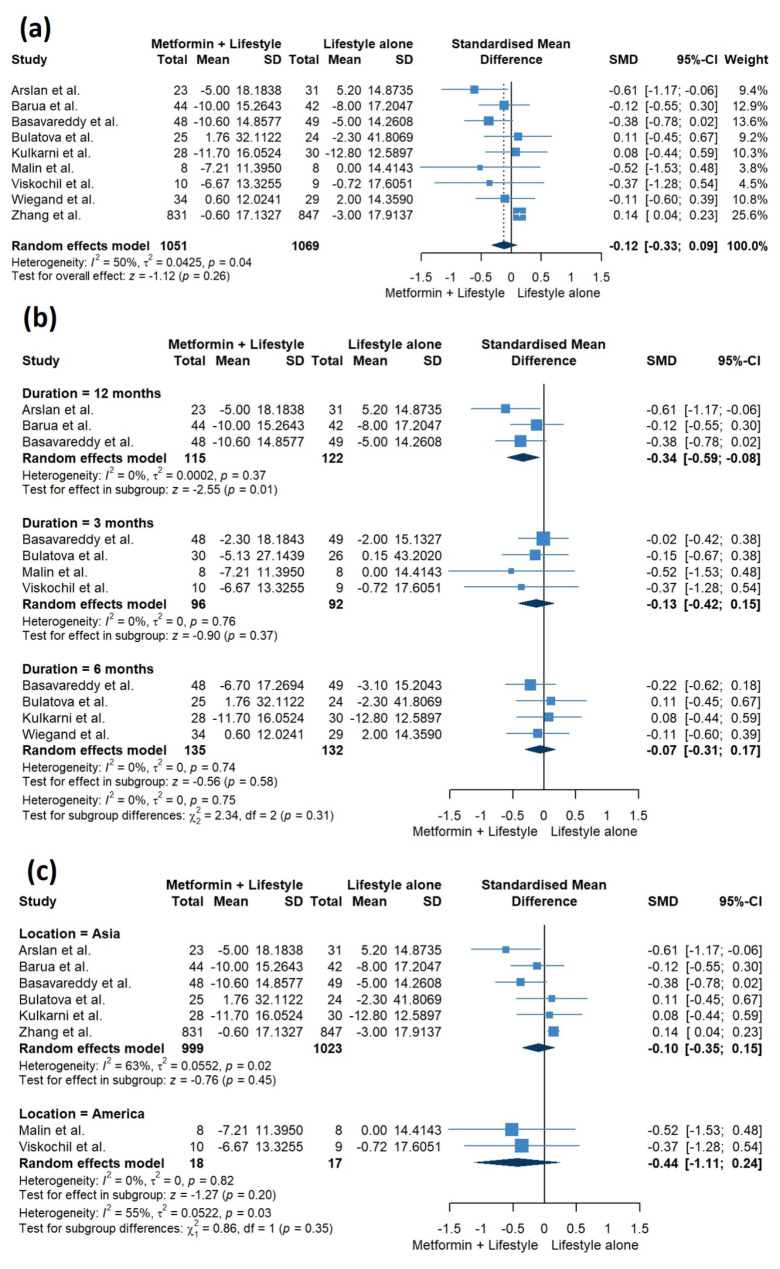
Pooled studies for fasting blood glucose levels **(a)** at endpoints, **(b)** with subgrouping based on the follow-up duration, **(c)** with subgrouping based on the geographical distribution

Our pooled analysis at the endpoints of included studies did not reveal any statistically significant difference between metformin plus lifestyle interventions versus lifestyle interventions alone (SMD = -0.12, 95% CI [-0.33, 0.09], *P* = 0.26) (Fig. 5a). The pooled studies were heterogenous (I^2^ = 50%, *P* = 0.04). To address this heterogeneity, we conducted a sensitivity analysis using the leave-one-out model, which showed that heterogeneity was best resolved by excluding Zhang et al., and the results became statistically significant, favoring the combination of metformin and lifestyle interventions (SMD = -0.21, 95% CI [-0.39, -0.02], I^2^ = 0%) (Supplementary Fig. S4). In contrast, sensitivity analysis omitting Wiegand et al. showed that the two groups were comparable (SMD = -0.13, 95% CI [-0.35, 0.10], I^2^ = 55%) (Supplementary Fig. S4).

Interestingly, our subgroup analysis based on different time points revealed that there was no significant difference between the two groups at both 3 and 6 months (SMD = -0.13, 95% CI [-0.42, 0.15], *P* = 0.37; SMD = -0.07, 95% CI [-0.31, 0.17], *P* = 0.58, respectively). However, adding metformin to lifestyle interventions significantly decreased FPG at 12 months, compared to lifestyle interventions alone (SMD = -0.34, 95% CI [-0.59, -0.08], *P* = 0.01). The studies within each subgroup exhibited homogeneity (I^2^ = 0%, *P* = 0.76; I^2^ = 0%, *P* = 0.74; I^2^ = 3%, *P* = 0.37, respectively) (Fig. 5b). Subgroup analysis based on the geographical distribution showed that the two groups were comparable in studies conducted in both Asia and America (SMD = -0.10, 95% CI [-0.35, 0.15], *P* = 0.45; SMD = -0.44, 95% CI [-1.11, 0.24], *P =* 0.20, respectively) (Fig. 5c).

#### Blood pressure measurements

##### Diastolic blood pressure

There was no statistically significant difference in DBP changes between the two groups (SMD = 0.04, 95% CI [-0.05, 0.13], *P* = 0.42). The pooled studies demonstrated homogeneity (I^2^ = 0%, *P* = 0.95) (Supplementary Fig. S5). The results remained non-significant after omitting Zhang et al. and Wiegand et al. (SMD = -0.04, 95% CI [-0.32, 0.24]; SMD = 0.04, 95% CI [-0.05, 0.13], respectively) (Table 3 and Supplementary Fig. S6).

##### Systolic blood pressure

Similarly, there was no statistically significant difference in SBP changes between the two groups (SMD = -0.04, 95% CI [-0.13, 0.05], *P* = 0.34). The pooled studies demonstrated homogeneity (I^2^ = 0%, *P* = 0.75) (Supplementary Fig. S7). The results remained non-significant after omitting Zhang et al. (SMD = -0.16, 95%CI [-0.41, 0.08], Table 3) and Supplementary Fig. S8) and Wiegand et al. (SMD = -0.04, 95% CI [-0.13, 0.05], Supplementary Fig. S8).

#### Body weight measurements

##### Body mass index

Our pooled analysis at the studies’ endpoints revealed that there was no statistically significant difference between the two groups in terms of BMI changes (SMD = -0.02, 95% CI [-0.10, 0.07], *P* = 0.67). The pooled studies demonstrated homogeneity (I^2^ = 0%, *P* = 0.80) (Supplementary Fig. S9). The results remained non-significant after omitting Zhang et al. (SMD = -0.03, 95% CI [-0.22, 0.17], Table 3 and Supplementary Fig. S10).

In addition, our subgroup analysis at 6 and 12 months showed similar results (SMD = -0.12, 95% CI [-0.42, 0.19], *P* = 0.45; SMD = 0.04, 95% CI [-0.22, 0.30], *P* = 0.76, respectively). The pooled studies within these subgroups displayed homogeneity (I^2^ = 0%, *P* = 0.41; I^2^ = 0%, *P* = 0.71, respectively) (Supplementary Fig. S11). Moreover, our subgroup analysis based on the age group revealed that adding metformin to lifestyle interventions was comparable to lifestyle interventions alone in both adults and adolescents (SMD = -0.01, 95% CI [-0.10, 0.08], *P* = 0.81; SMD = -0.17, 95% CI [-0.66, 0.31], *P* = 0.51, respectively) (Supplementary Fig. S12).

##### Waist circumference

Adding metformin to lifestyle interventions showed comparable results to lifestyle interventions alone in terms of changes in waist circumference (SMD = 0.00, 95% CI [-0.09, 0.10], *P* = 0.93). The pooled studies demonstrated homogeneity (I^2^ = 0%, *P* = 0.57) (Supplementary Fig. S13). The results remained non-significant after omitting Zhang et al. (Table 3 and Supplementary Fig. S14).

##### Weight

Our pooled studies at endpoints showed a trend towards higher weight with lifestyle interventions plus metformin; however, this did not reach statistical significance (SMD = 0.12, 95% CI [-0.03, 0.27], *P* = 0.11). The pooled studies demonstrated heterogeneity (I^2^ = 57%, *P* = 0.07) (Supplementary Fig. S15). To address this heterogeneity, we conducted a sensitivity analysis using the leave-one-out model, which showed that it was best resolved by excluding Zhang et al., and the results became significant favoring the lifestyle interventions only group (SMD = 0.22, 95% CI [0.10, 0.33], I^2^ = 0%) (Supplementary Fig. S16).

#### Insulin resistance

Our pooled analysis at the studies endpoints showed no statistically significant difference between the two groups, with a SMD − 0.06 (95% CI [-0.16, 0.03], *P* = 0.17). The pooled studies demonstrated homogeneity (I^2^ = 0%, *P* = 0.86) (Supplementary Fig. S17). The results remained non-significant after omitting Zhang et al. and Wiegand et al. (SMD = -0.15, 95% CI [-0.51, 0.22]; SMD = -0.06, 95% CI [-0.16, 0.03], respectively) (Table 3 and Supplementary Fig. S18).

#### Lipid profile measurements

##### Serum HDL

Our pooled analysis at 6 and 12 months showed no statistically significant difference between the two groups (SMD = 0.03, 95% CI [-0.27, 0.33], *P* = 0. 83; SMD = -0.02, 95% CI [-0.28, 0.23], *P* = 0.87, respectively). The pooled studies at 6 and 12 months were homogenous (I^2^ = 0%, *P* = 0.80; I^2^ = 0%, *P* = 0.95) (Supplementary Fig. S19). The results remained statistically non-significant after omitting the study by Wiegand et al. (SMD = 0.02, 95% CI [-0.20, 0.23], Supplementary Fig. S20).

##### Serum LDL

Our pooled analysis at 6 and 12 months showed no statistically significant difference between the two groups (SMD = 0.30, 95% CI [-0.01, 0.59], *P* = 0.05; SMD = -0.02, 95% CI [-0.27, 0.24], *P* = 0.89, respectively). The pooled studies within each subgroup displayed homogeneity (I^2^ = 0%, *P* = 0.58; I^2^ = 0%, *P* = 0.75, respectively) (Supplementary Fig. S21). The results remained non-significant after omitting Wiegand et al. (SMD = 0.06, 95% CI [-0.15, 0.27], Supplementary Fig. S22).

##### Total cholesterol

Our pooled analysis at 6 and 12 months showed that the changes in total cholesterol were comparable between the two groups (SMD = 0.10, 95% CI [-0.20, 0.40], *P* = 0.52; SMD = -0.02, 95% CI [-0.28, 0.23], *P* = 0.85, respectively). The pooled studies displayed homogeneity (I^2^ = 0%, *P* = 0.49; I^2^ = 0%, *P* = 0.57, respectively) (Supplementary Fig. S23). The results remained non-significant after omitting Wiegand et al. (SMD = 0.03, 95% CI [-0.18, 0.24], Supplementary Fig. S24).

##### Serum triglycerides

Our pooled analysis at 6 and 12 months showed that the changes in serum triglycerides were comparable between the two groups (SMD = -0.02, 95% CI [-0.22, 0.17], *P* = 0.80; SMD = 0.05, 95% CI [-0.21, 0.30], *P* = 0.71, respectively). The pooled studies displayed homogeneity (I^2^ = 17%, *P* = 0.30; I^2^ = 0%, *P* = 0.46, respectively) (Supplementary Fig. S25).

**Table 3:** Summary of our analysis

Outcome		Analysis or subgroup	Number of pooled RCTs	Estimate	95% CI	*P* value	Heterogeneity *P* value	Estimate after omitting Zhang et al.
**Glycemic control**	T2DM	at endpoints	5	0.85	[0.75, 0.97]	0.01	0.7	1 [0.75, 1.34]
	FPG	at 3 months	4	-0.13	[-0.42, 0.15]	0.37	0.76	-
		at 6 months	4	-0.07	[-0.31, 0.17]	0.58	0.75	-
		at 12 months	3	-0.34	[-0.59, -0.08]	0.01	0.37	-
		at endpoints	9	-0.12	[-0.33, 0.09]	0.26	0.04*	-0.21 [-0.39, -0.02]
	HbA1c	at 3 months	2	-0.37	[-0.68, -0.05]	0.02	0.97	-
		at 6 months	3	-0.35	[-0.62, -0.07]	0.01	0.7	-
		at 12 months	2	-0.2	[-0.49, 0.09]	0.17	0.86	-
		at endpoints	5	-0.1	[-0.19, -0.01]	0.03	0.89	-0.21 [-0.44, 0.02]
**Plasma lipids**	Triglycerides	at 6 months	3	-0.12	[-0.45, 0.20]	0.46	0.3	-
		at 12 months	3	0.05	[-0.21, 0.30]	0.71	0.46	-
		Overall	6	-0.02	[-0.22, 0.17]	0.8	0.45	-
	Total cholesterol	at 6 months	3	0.1	[-0.20, 0.40]	0.52	0.49	-
		at 12 months	3	-0.02	[-0.28, 0.23]	0.85	0.57	-
		Overall	6	0.03	[-0.17, 0.22]	0.79	0.71	-
	Serum HDL	at 6 months	3	0.03	[-0.27, 0.33]	0. 83	0.8	-
		at 12 months	3	-0.02	[-0.28, 0.23]	0.87	0.95	-
		Overall	6	0	[-0.19, 0.19]	0.99	0.99	-
	Serum LDL	at 6 months	3	0.3	[0.00, 0.59]	0.05	0.58	-
		at 12 months	3	-0.02	[-0.27, 0.24]	0.89	0.75	-
		Overall	6	0.11	[-0.08, 0.31]	0.25	0.53	-
**Body weight**	Weight	at endpoints	4	0.12	[-0.03, 0.27]	0.11	0.07	0.22 [0.10, 0.33]
	BMI	at 6 months	3	-0.12	[-0.42, 0.19]	0.45	0.41	-
		at 12 months	3	0.04	[-0.22, 0.30]	0.76	0.71	-
		at endpoints	7	-0.02	[-0.10, 0.07]	0.67	0.8	-0.03 [-0.22, 0.17]
	WC	at endpoints	3	0	[-0.09, 0.10]	0.93	0.57	0.12 [-0.29, 0.53]
**Blood pressure**	SBP	at endpoints	5	-0.04	[-0.13, 0.05]	0.34	0.75	-0.16 [-0.41, 0.08]
	DBP	at endpoints	4	0.04	[-0.05, 0.13]	0.42	0.95	-0.04 [-0.32, 0.24]
**Insulin resistance**	HOMA-IR	at endpoints	3	-0.06	[-0.16, 0.03]	0.17	0.86	-0.15 [-0.51, 0.22]

T2DM, type 2 diabetes mellitus; FPG, fasting plasma glucose; HbA1c, glycated hemoglobin; HDL, high density lipoprotein; LDL, low density lipoprotein; BMI, body mass index; WC, waist circumference; SBP, systolic blood pressure; DBP, diastolic blood pressure; CI, confidence interval. * Heterogeneity best resolved by omitting Zhang et al.

## Discussion

### Summary of the findings

In our meta-analysis, adding metformin to lifestyle interventions significantly reduced HbA1c levels and the incidence of type 2 diabetes at the endpoints of the included studies. Interestingly, adding metformin to lifestyle interventions was comparable to lifestyle interventions alone in terms of FPG changes at both 3 and 6 months; however, it significantly reduced FPG at 12 months. In addition, adding metformin to lifestyle interventions significantly decreased HbA1c at 3 and 6 months, compared to the lifestyle interventions alone. However, the pooled studies at 12 months indicated no significant difference between the two groups. In summary, adding metformin to lifestyle interventions showed late-onset rapid glucose control evidenced by FPG, which started only after 12 months of consistent therapy. In contrast, delayed glucose control evidenced by HbA1c started early but for a short-term. Finally, our analysis found no significant differences between the two groups in terms of plasma lipids, blood pressure, body weight, and insulin resistance measurements. Together, this highlights that adding metformin to lifestyle interventions may be superior to lifestyle interventions alone in reducing HbA1c and FPG levels in individuals with prediabetes, and consequently, may reduce the risk of progression to diabetes in these individuals. However, this combined approach appears to be limited in terms of all our secondary outcomes. Finally, a longer duration of this combined approach may be required to observe the desired effects.

### Explanation of the findings

The observed significant reduction in the incidence of type 2 diabetes and glycemic control parameters in our meta-analysis may be attributed to the synergistic effects targeting multiple pathways implicated in diabetes progression. Lifestyle interventions, encompassing dietary modifications and increased physical activity, have a well-established track record in preventing or delaying the onset of type 2 diabetes [13, 14]. When combined with metformin, an oral anti-diabetic medication addressing insulin resistance and hepatic glucose production, this amalgamation may provide a more effective strategy [49].

Metformin contributes to long-term glycemic control by enhancing insulin action and decreasing glucose production in the liver, resulting in diminished HbA1c levels, which measure the average blood sugar levels over several months [49]. This measure of average blood sugar levels over several months indicates sustained improvement and consequently mitigates the risk of diabetes-related complications.

In addition, the combined approach’s efficacy in short-term glucose regulation, evidenced by decreased fasting blood glucose levels, may be attributed to the impact of lifestyle interventions and metformin’s insulin-sensitizing properties [49, 50]. This dual mechanism addresses both immediate and prolonged aspects of glycemic control, offering a comprehensive strategy for the prevention and management of diabetes.

The absence of a significant difference between the two groups in terms of HbA1c levels at 12 months may be attributed to several factors. First, the intervention’s efficacy may diminish over time. In the initial phases, patients may be more compliant with lifestyle modification and medication use, potentially leading to more substantial early improvements in glucose regulation. Over time, adherence may wane, and the body may adapt to the intervention, ultimately plateauing the effects. Additionally, variations in the specific components of lifestyle interventions can contribute to these varied outcomes. In contrast, the absence of a significant difference in FPG levels at 3 and 6 months with the observed significant reduction in the metformin plus lifestyle interventions group at 12 months implies that while the immediate impact on fasting blood glucose might not be pronounced, a longer intervention duration may be necessary to observe the desired effects.

While the combined approach demonstrates efficacy in glycemic control, its impact on other parameters may be limited. This underscores the complexity of diabetes management and highlights the importance of tailoring interventions to address the multifaceted nature of the condition.

We believe that Zhang et al.‘s study significantly influences our meta-analysis, being the largest included RCT, comprising 1678 participants [18]. In contrast, other included RCTs had 16 to 262 participants, making them more susceptible to type 2 errors. In addition, Zhang et al.‘s study had a longer follow-up duration compared to most included RCTs. These variations underscore the importance of recognizing Zhang et al.‘s unique contributions in our analysis [18].

### Agreements and disagreements with previous studies

A previous meta-analysis in 2019 aimed to comprehensively evaluate the preventive efficacy of metformin on type 2 diabetes in high-risk prediabetic populations. Metformin was compared with multiple other pharmacological or non-pharmacological interventions including standard or intensive diet and exercise. Although metformin resulted in reduced incidence of type 2 diabetes and FPG when compared to standard diet and exercise with or without placebo based on 12 and 15 RCTs, respectively, no advantage was noticed on both incidence of type 2 diabetes and FPG when metformin with or without intensive diet and exercise was compared to intensive diet and exercise alone. However, it is to be noted that the evidence for the comparison of metformin combined with intensive diet and exercise to intensive diet and exercise alone was derived from two RCTs only and was judged to have very low-quality evidence [23]. Moreover, the meta-analysis did not compare metformin with standard diet and exercise regimen to standard diet and exercise regimen only.

Other drugs have been tested for preventing the onset of type 2 diabetes, such as voglibose [51], DPP-4 inhibitors [52], orlistat [53], and valsartan [54]. In a clinical trial conducted by Kawamori et al., individuals undergoing diet and exercise along with voglibose had a significantly lower risk of progressing to type 2 diabetes compared to those on a placebo. A greater number of individuals in the voglibose group achieved normoglycemia, although adverse events were more prevalent in this group, including serious events like cholecystitis, colonic polyp, rectal neoplasm, inguinal hernia, liver dysfunction, and subarachnoid hemorrhage [51]. Similarly, a longitudinal cross-sectional study on patients recruited from the diabetes prevention trial (PRELLIM) comparing metformin alone to metformin and linagliptin showed favorable outcomes of the combination on insulin sensitivity and pancreatic function [52]. Likewise, a recent meta-analysis showed that orlistat can control weight, reduce FPG, and subsequently, delay the progression to diabetes [53]. Finally, studies suggest that inhibition of the renin–angiotensin system, including ACE inhibitors and ARBs like valsartan, may reduce diabetes incidence and cardiovascular risk [54]. The utilization of valsartan over 5 years, alongside lifestyle modifications, resulted in a relative reduction of 14% in the incidence of diabetes. However, it is noteworthy that valsartan, when compared to placebo, did not demonstrate a significant reduction in the incidence of extended cardiovascular outcomes.

### Strength points and limitations

To date, our study is the most comprehensive meta-analysis comparing the effects of adding metformin to lifestyle interventions versus lifestyle interventions alone. We included published RCTs only to provide strong evidence. We examined a diverse range of outcomes, including both glycemic and metabolic parameters. In addition, we performed a sensitivity analysis to assess the robustness of our findings. Furthermore, we examined our outcomes at different time points by performing subgroup analysis based on the follow-up duration. It is imperative to note that we exclusively included studies in the English language. Additionally, our meta-analysis is limited by the small number of pooled studies in most outcomes. Therefore, we could not assess the risk of publication bias using Egger’s et al. test. Furthermore, most included studies are from Asian countries. Therefore, healthcare providers from other countries should interpret our findings with caution. The intensity of lifestyle interventions among the included RCTs ranged from moderate to high intensity. In addition, some included RCTs did not provide sufficient information regarding the intensity of such interventions. Finally, we recognize the substantial influence of Zhang et al., who emerged as a crucial contributor to our meta-analysis.

### Implications of our findings in practice

Healthcare practitioners should consider integrating both pharmacological and lifestyle interventions, recognizing the synergistic benefits they offer. However, the observed lack of significant impact on various metabolic and cardiovascular parameters emphasizes the complexity of diabetes management. Therefore, it is crucial to tailor interventions based on individual patient characteristics, considering factors such as age, baseline BMI, and glycemic status. Moreover, the accurate therapeutic dose of metformin that balances efficacy with the least side effect profile based on variables like body weight should be taken into consideration [55].

### Recommendations

We recommend future well-designed RCTs to assess the sustainability of the observed effects over extended periods. Diverse population inclusion is also recommended to understand the intervention’s effectiveness across various demographic groups. The required dose of metformin to maintain sustainable outcomes regarding efficacy and safety alongside lifestyle interventions is another important area. Furthermore, other recently proposed adjuncts to improve metformin efficacy for diabetes prevention including controlling the gut microbiota [56] should be investigated. Finally, investigating the underlying mechanisms of treatment responses and exploring the influence of intervention duration are essential for optimizing interventions and may contribute to more comprehensive and personalized approaches to managing prediabetes.

## Conclusion

In conclusion, adding metformin to lifestyle interventions may be superior to lifestyle interventions alone in reducing HbA1c and FPG levels in individuals with prediabetes, and consequently, may reduce the risk of progression to diabetes in these individuals. However, this combined approach appears to be limited in terms of lipid profile, blood pressure, and body weight measurements. Future well-designed RCTs are required to confirm our findings.

## Electronic supplementary material

Below is the link to the electronic supplementary material.

Supplementary Material 1

Supplementary Material 2

## Data Availability

The datasets used and/or analyzed during the current study are available as MS Excel files (.xlsx) from the corresponding author upon reasonable request.

## Abbreviations

2hPG: 2-hour plasma glucose level after a 75-g oral glucose challenge
RCTs: Randomized controlled trials
PRISMA: Preferred reporting items for systematic reviews and meta-analysis
RR: Relative risk
CI: Confidence interval
SMD: Standardized mean difference
ROB2: Risk of bias 2
IGT: Impaired glucose tolerance
IFG: Impaired fasting glucose
USA: United States of America
LSI: Lifestyle intervention
BMI: Body mass index
FPG: Fasting plasma glucose
HbA1c: Glycated hemoglobin
