# Supplementary material for: Metformin plus lifestyle interventions versus lifestyle interventions alone for the delay or prevention of type 2 diabetes in individuals with prediabetes: a meta-analysis of randomized controlled trials
Source: Diabetol Metab Syndr. 2024 Nov 14;16:273. doi: 10.1186/s13098-024-01504-8 (PMC11562588; doi:10.1186/s13098-024-01504-8)
Supplement: Supplementary file 1 — Supplementary Material 1 [file 13098_2024_1504_MOESM1_ESM.docx]

**Supplementary materials**

**Supplementary Table 1** Search terms and results in different databases.

| **Database** | **Search strategy** | **Filter** | **Results** | **Search date** |
| --- | --- | --- | --- | --- |
| PubMed | (lifestyle AND (Metformin OR Dimethylbiguanidine OR Dimethylguanylguanidine OR Glucophage) AND (prediabetes OR pre-diabetes OR prediabetes OR "glucose intolerance" OR "impaired fasting glucose" OR "impaired glucose metabolism" OR "impaired glucose tolerance" OR IFG OR IGT)) | All Fields | 510 | March 20, 2024 |
| Web of Science |  | Topic | 599 |  |
| Scopus |  | Article title, Abstract, Keywords | 1611 |  |
| Cochrane |  | Title, Abstract, Keyword | 301 |  |

**Supplementary Table 2** Baseline characteristics of patients in each included study.

| **Study ID** | **Group** | **Sample size** | **Age (Years) Mean ± SD** | **BMI (Kg/m2) Mean ± SD** | **HbA1c (%) Mean ± SD** | **FPG (mg/dl) Mean ± SD** | **PG2h (mmol/L) Mean ± SD** |
| --- | --- | --- | --- | --- | --- | --- | --- |
| Arslan et al. [1] | Met + LSI | 23 | 44.9 ± 8.7 | 32.9 ± 5.3 | NR | 99.5 ± 13.3 | NR |
|  | LSI | 31 |  | 32.5 ± 3.5 | NR | 103.1 ± 11.1 | NR |
| Barua et al. [2] | Met + LSI | 50 | 45.2 ± 5.9 | 24.5 ± 7.1 | 5.81 ± 0.47 | 111 ± 13 | 8.71 ± 1.44 |
|  | LSI | 50 | 44.5 ± 6.4 | 24.1 ± 6.7 | 5.91 ± 0.44 | 109 ± 14 | 8.82 ± 1.39 |
| Basavareddy et al. [3] | Met + LSI | 53 | 46.57 ± 9.65 | 26.96 ± 3.74 | 6.25 ± 0.21 | 107.89 ± 12.39 | NR |
|  | LSI | 51 | 48 ± 9.04 | 26.81 ± 2.96 | 6.15 ± 0.22 | 104 ± 13.1 | NR |
| Bulatova et al. [4] | Met + LSI | 26 | 50.70 ± 1.86 | 36.98 ± 1.57 | 6.30 ± 0.39 | 108.00 ± 20.90 | NR |
|  | LSI | 27 | 50.70 ± 1.78 | 39.60 ± 1.58 | 6.06 ± 0.36 | 110.97 ± 32.22 | NR |
| Hydrie et al. [5] | Met + LSI | 95 | 43.5 ± 8.4 | 28.1 ± 4.3 | NR | NR | NR |
|  | LSI | 114 | 43.1 ± 10.1 | 26.1 ± 4.7 | NR | NR | NR |
| Kulkarni et al. [6] | Met + LSI | 35 | 49.4±9.2 | 28.1±4.9 | 6.1±0.23 | 108.9±8.2 | NR |
|  | LSI | 35 | 45.3±10.9 | 29.3±3.8 | 6.13±0.27 | 109.4±6.3 | NR |
| Love-Osborne et al. [7] | Met + LSI | 60 | 15.5 ± 1.7 | 39.4 ± 6.5 | NR | 88.5 ± 11.6 | 6.22 ± 1.25 |
|  | LSI | 25 | 14.2 ± 4.6 | 39.3 ± 7.2 | NR | 88.8 ± 10.0 | 6.13 ± 1.47 |
| Malin et al. [8] | Met + LSI | 8 | 49.1 ± 6.6 | 31.2 ± 5.3 | NR | 104.5 ± 10.19 | 9.5 ± 1.7 |
|  | LSI | 8 | 45.4 ± 8.0 | 33.5 ± 4.1 | NR | 95.5 ± 10.2 | 10.2 ± 1.0 |
| Ramachandran at al. [9] | Met + LSI | 129 | 46.3±5.7 | 25.6 ± 3.3 | 6.2 ± 0.6 | 97.3 ± 14.41 | 8.5±0.7 |
|  | LSI | 133 | 46.1±5.7 | 25.7 ± 3.3 | 6.1 ± 0.5 | 97.3 ± 12.61 | 8.5±0.7 |
| Viskochil et al. [10] | Met + LSI | 10 | 49.5 ± 1.8 | NR | NR | 106.3 ± 3.6 | 9.8 ± 0.5 |
|  | LSI | 9 | 46.2 ± 2.6 | NR | NR | 99.1 ± 5.41 | 10.4 ± 0.3 |
| Wiegand et al. [11] | Met + LSI | 36 | 13.77 ± 2.13 | 34.25 ± 4.95 | NR | 94 ± 8.3 | 6.65 ± 0.95 |
|  | LSI | 34 |  | 35.47 ± 5.77 | NR | 95.3 ± 12.7 | 6.22 ± 1.22 |
| Zhang et al. [12] | Met + LSI | 831 | 52.33 ± 10.4 | 26.27 ± 2.88 | 5.86 ± 0.44 | 105.95 ± 11.35 | 8.94 ± 1.34 |
|  | LSI | 847 | 52 ± 9.65 | 26.28 ± 2.81 | 5.90 ± 0.41 | 106.49 ± 10.8 | 8.96 ± 1.32 |

BMI, body mass index; FPG, fasting plasma glucose; HbA1c, glycated hemoglobin; 2hPG, 2-hour plasma glucose level after a 75-g oral glucose challenge; Met, metformin; LSI, lifestyle interventions; NR, not reported.

**Supplementary Table 3** Lifestyle interventions incorporated by the included studies.

| **Study ID** | **Lifestyle intervention** | **Metformin** |
| --- | --- | --- |
| Arslan et al. [1] | Moderate-intensity physical activity at least 150 minutes/week and 7 % weight loss, as recommended by the American Diabetes Association. | 2000 mg/day |
| Barua et al. [2] | The participants received detailed, individualized counseling regarding their diet and exercise. Patients were followed-up through continuous visits. The patient who could not come physically was contacted over mobile phone. | 500 mg open level drug |
| Basavareddy et al. [3] | The participants were counseled for intensive dietary habits, physical activity (walking for 30 minutes per day for 5 days/week), and drug intake. Participants were given a chart to record their adherence to these interventions. Patients were requested to bring this chart at each follow-up visit. | 500 mg once daily |
| Bulatova et al. [4] | Regular daily dietary intake (1200 calories/day for women and 1500 calories/day for men) alongside daily moderate exercise for 30 minutes to 1 hour. | 850 mg three times daily |
| Hydrie et al. [5] | Individually tailored diet control and moderate physical exercise for at least 30 minutes/day. The subjects were followed-up every two months by a dietician and a physical trainer to improve their physical fitness. | 500 mg twice daily |
| Kulkarni et al. [6] | Moderate intensity activity and dietary changes, which were reinforced by the healthcare facilitator through weekly standardized short message service and monthly phone calls. | 500 mg twice daily |
| Love-Osborne et al. [7] | Regular aerobic exercise and dietary changes with monthly follow-up visits. | 500 mg once daily then 500 mg twice daily and finally, 850 mg twice daily |
| Malin et al. [8] | low-fat diet combined with aerobic and resistance exercise 3 days a week for 60–75 minutes. | 2000 mg/day |
| Ramachandran et al. [9] | Diet modification and regular physical activity with regular follow-up through monthly phone conversations and in-person meetings. | 500 mg twice daily then 250 mg twice daily |
| Viskochil et al. [10] | 225 total minutes of supervised aerobic and resistance exercise training 3 days/week. | 1000 mg twice daily |
| Wiegand et al. [11] | Specialized diet control and sport classes with reinforcement sessions every 4–8 weeks. | 500 mg twice/day |
| Zhang et al. [12] | Intensive diet control combined with at least 30 minutes of physical exercises daily (5 days per week). | 850 mg once per day then 850 mg twice per day |


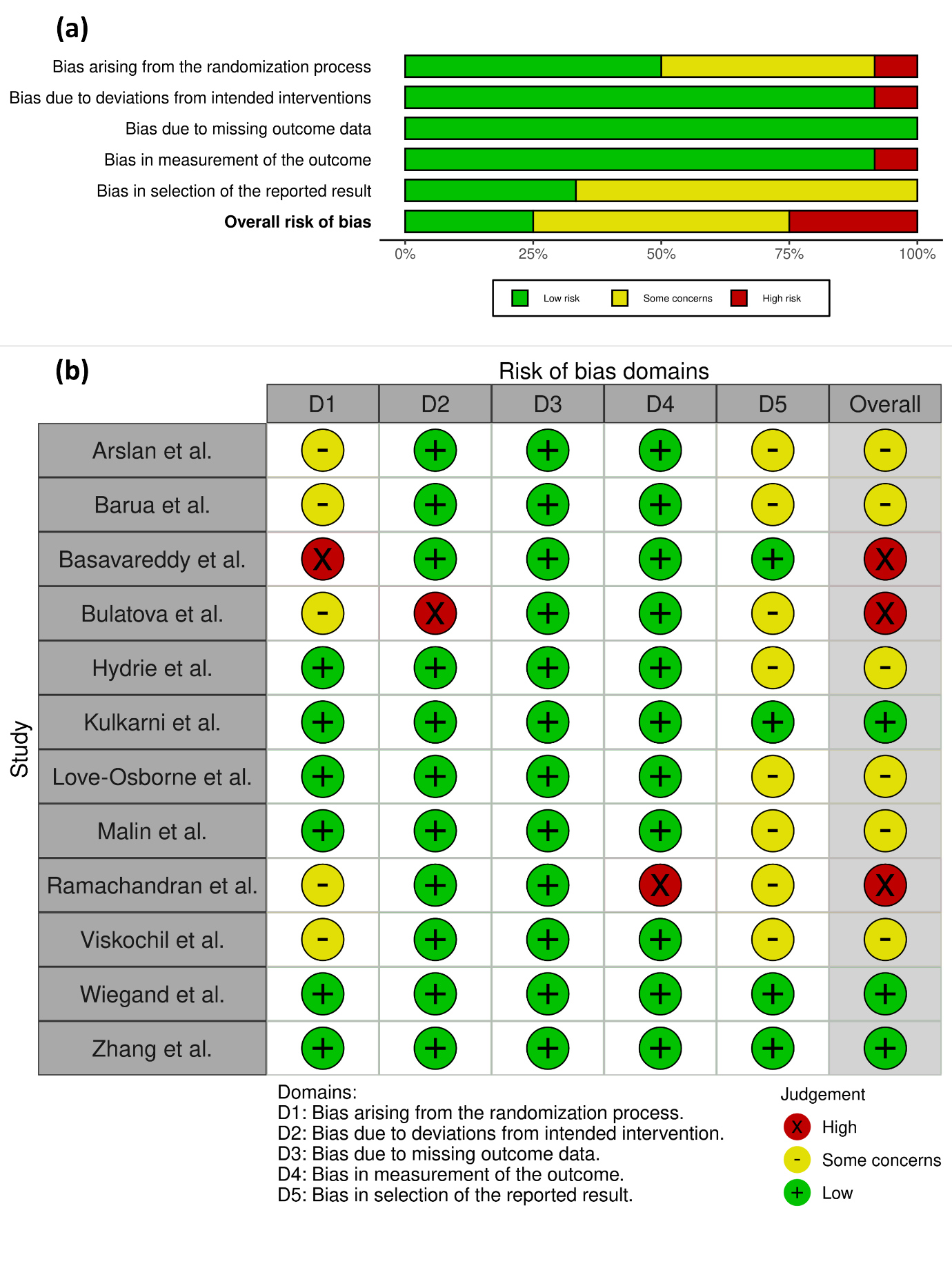
**Supplementary Fig. S1** **(a)** Risk of bias summary **(b)** Risk of bias graph.

**
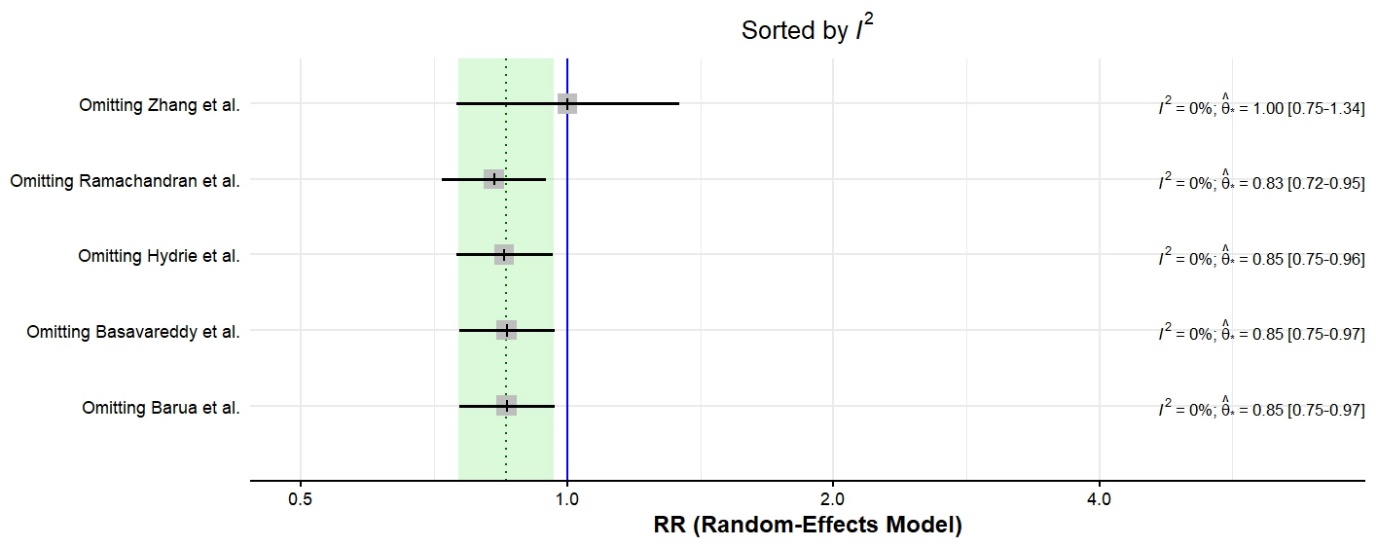
**

**Supplementary Fig. S2** Sensitivity analysis for the incidence of type 2 diabetes using the leave-one-out model.

**
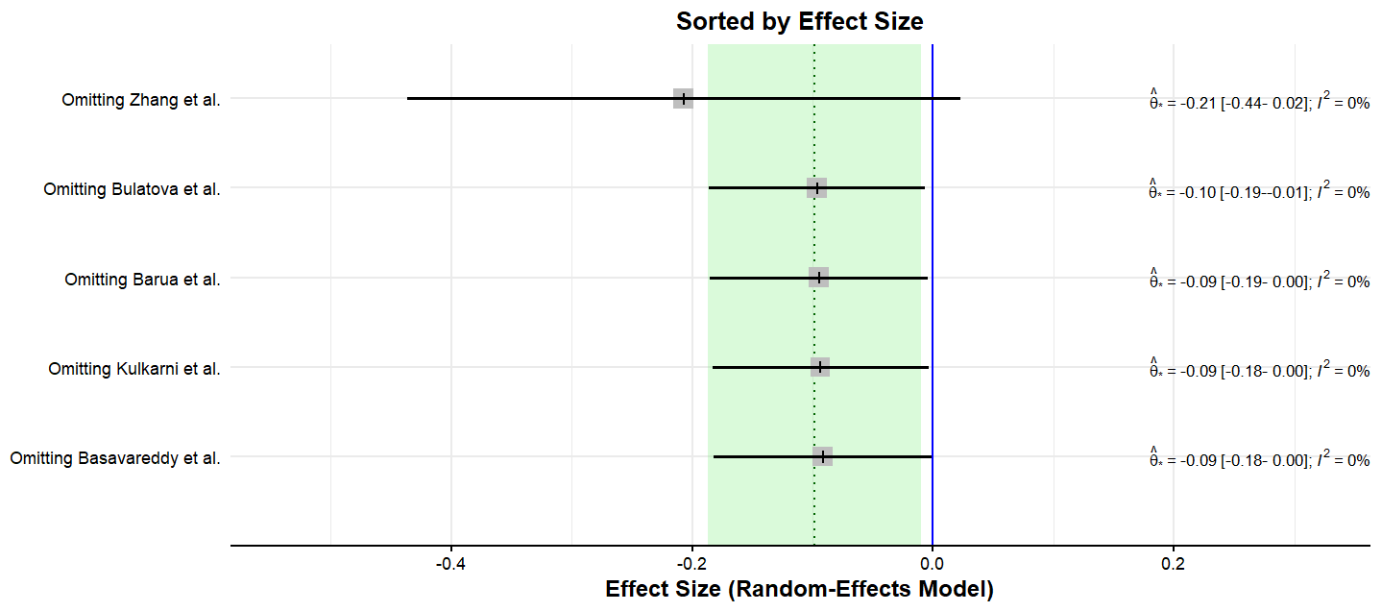
**

**Supplementary Fig. S3** Sensitivity analysis for HbA1c using the leave-one-out model.


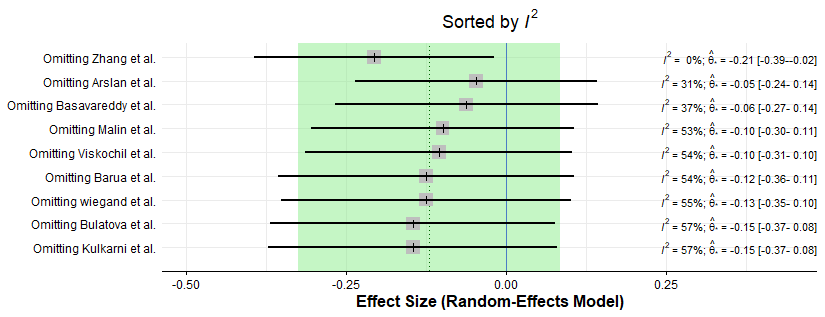


**Supplementary Fig. S4** Sensitivity analysis for fasting plasma glucose using the leave-one-out model.


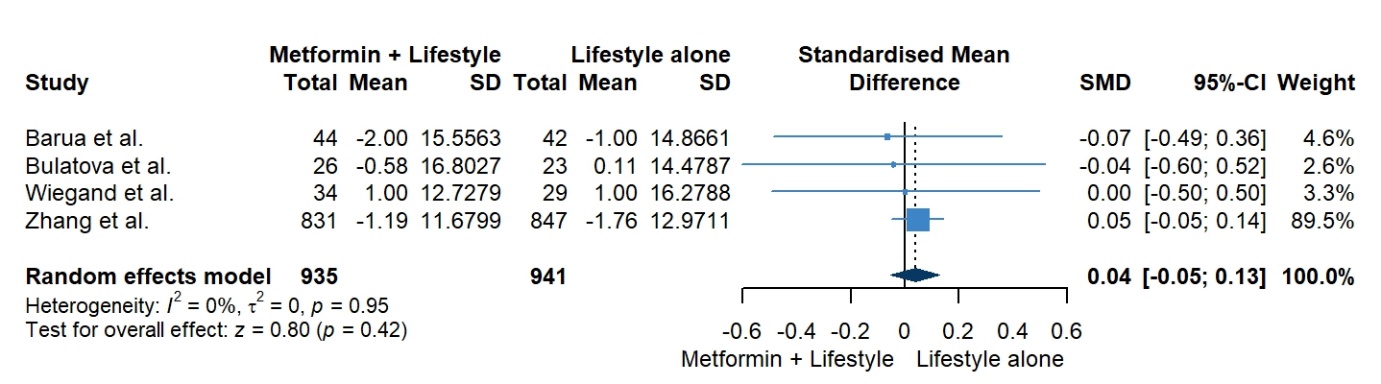


**Supplementary Fig. S5** Pooled studies for diastolic blood pressure.


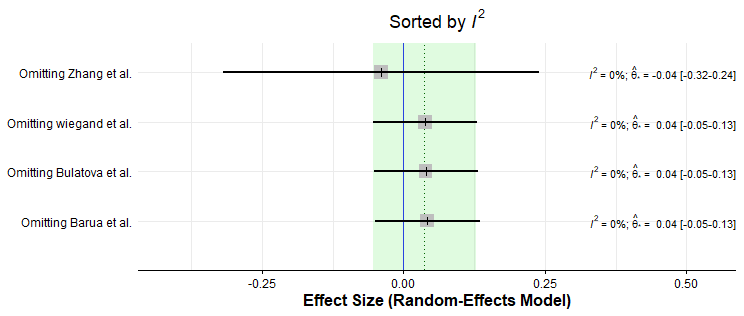


**Supplementary Fig. S6** Sensitivity analysis for diastolic blood pressure using the leave-one-out model.


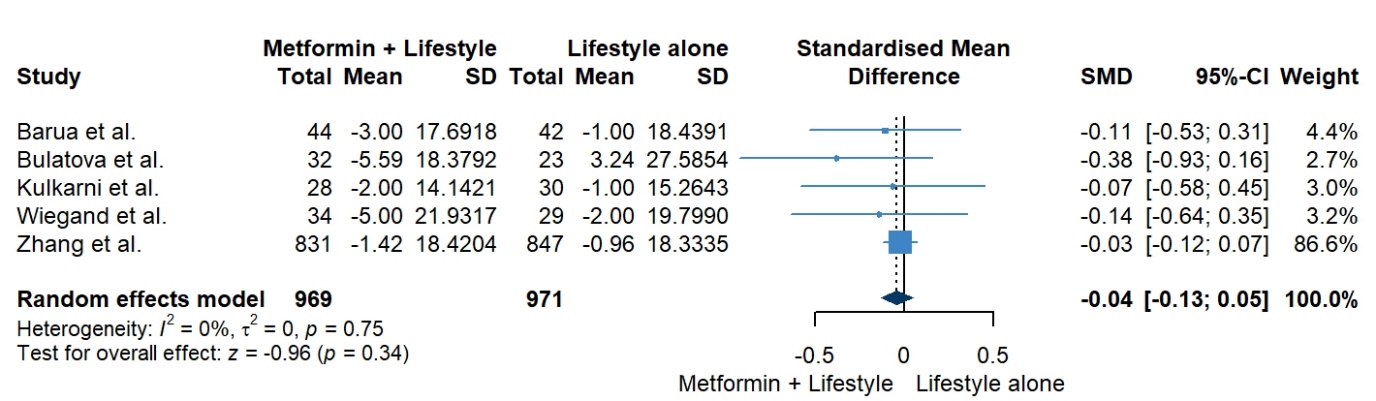


**Supplementary Fig. S7** Pooled studies for systolic blood pressure.


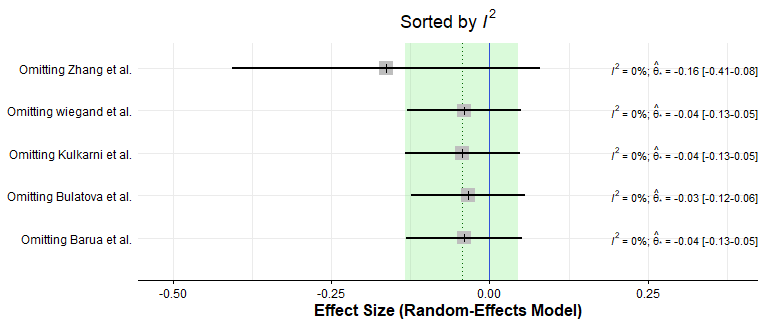


**Supplementary Fig. S8** Sensitivity analysis for systolic blood pressure using the leave-one-out model.


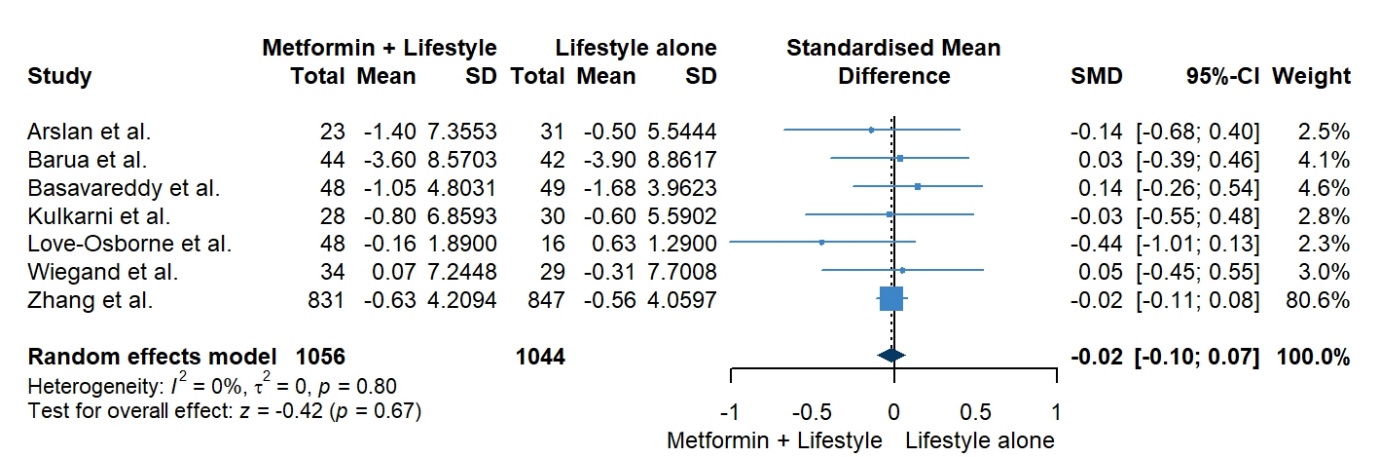


**Supplementary Fig. S9** Pooled studies for body mass index.


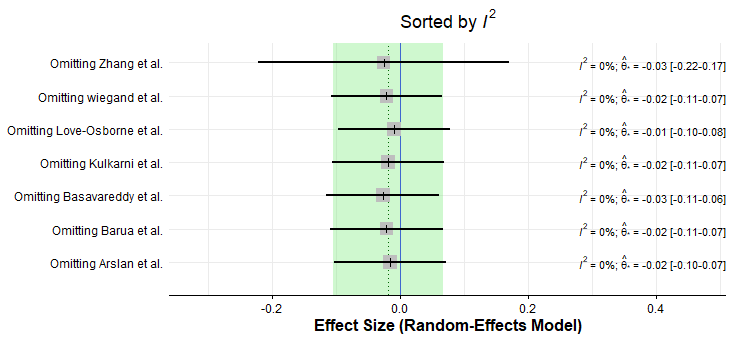


**Supplementary Fig. S10** Sensitivity analysis for body mass index using the leave-one-out model.


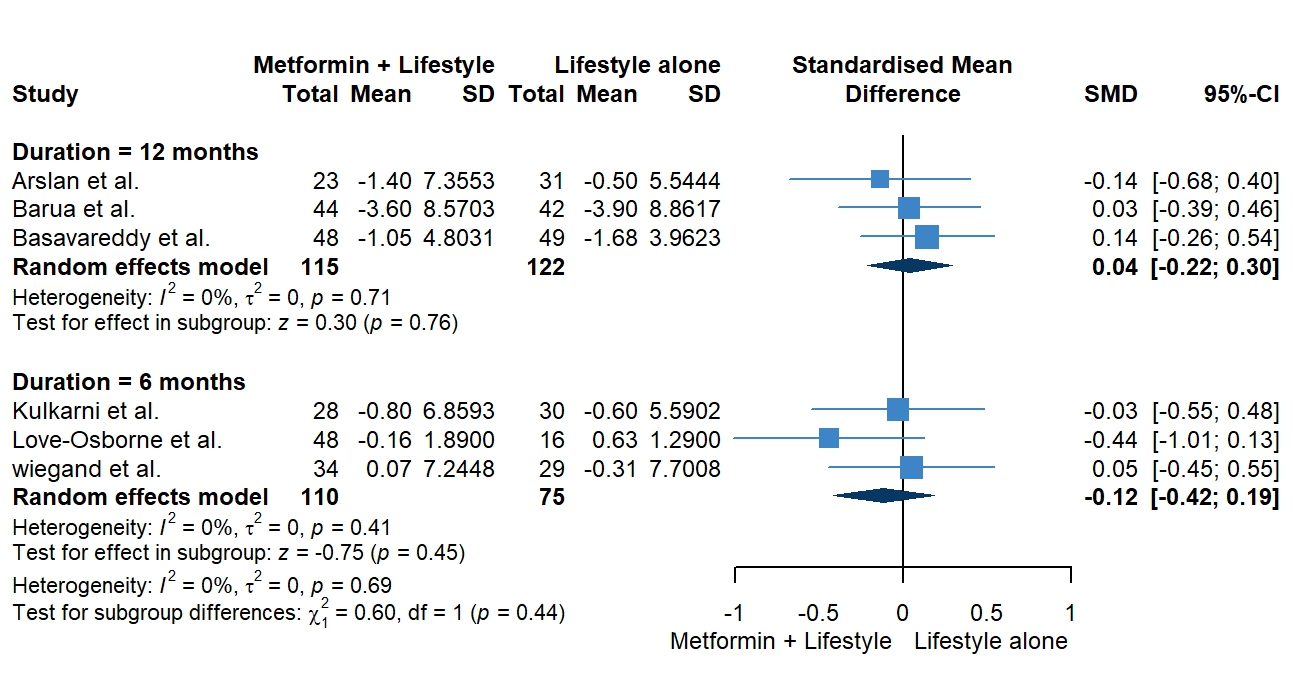


**Supplementary Fig. S11** Pooled studies for body mass index at 6 and 12 months.


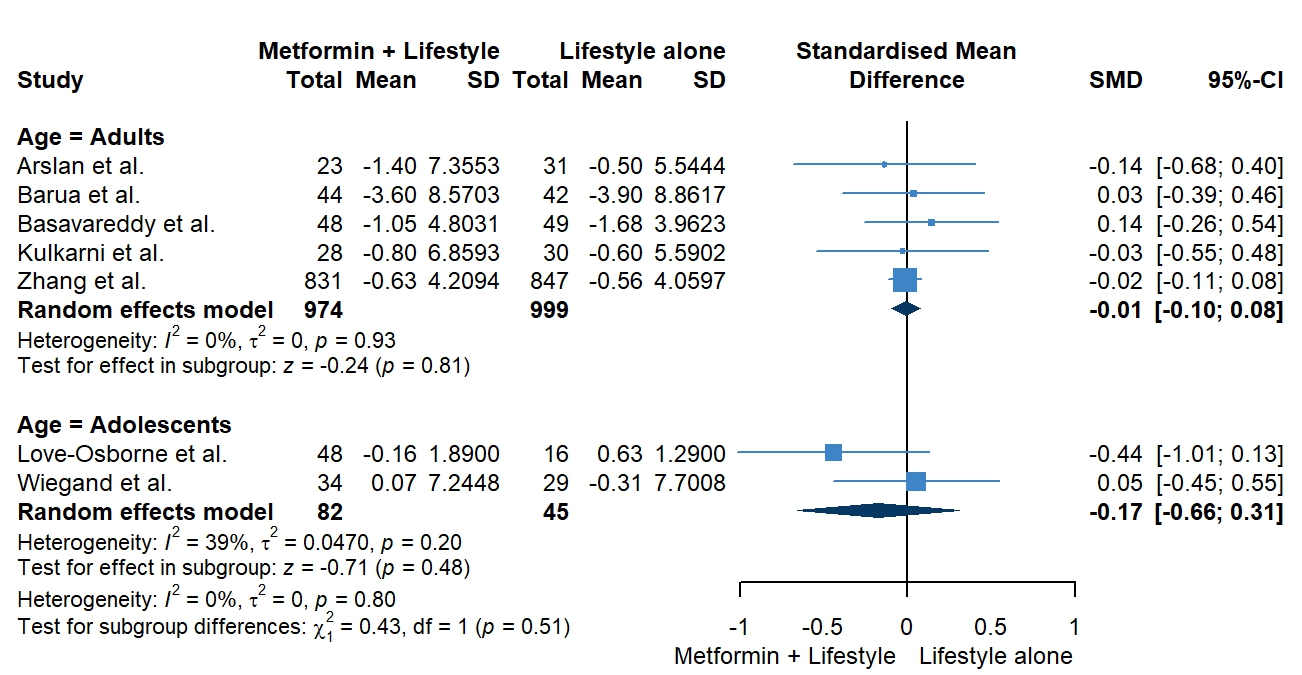


**Supplementary Fig. S12** Pooled studies for body mass index with subgrouping based on the age group.


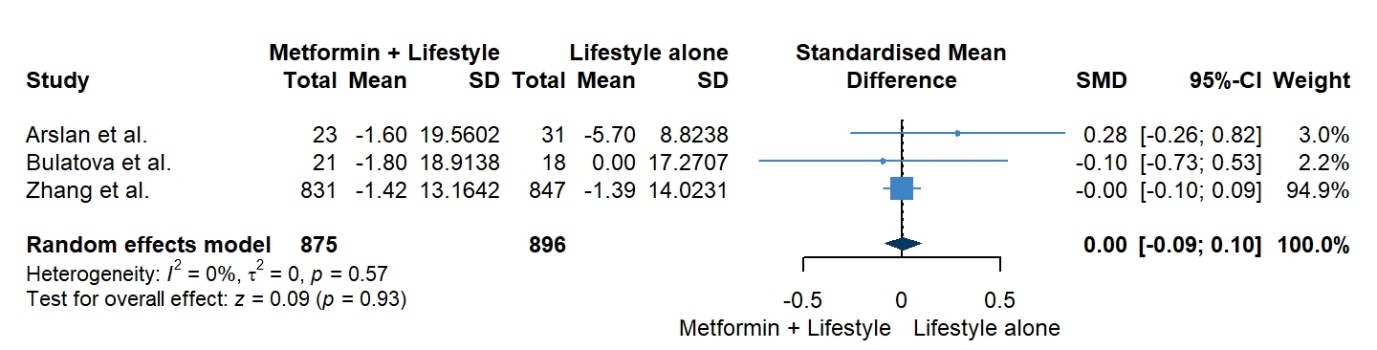


**Supplementary Fig. S13** Pooled studies for waist circumference.


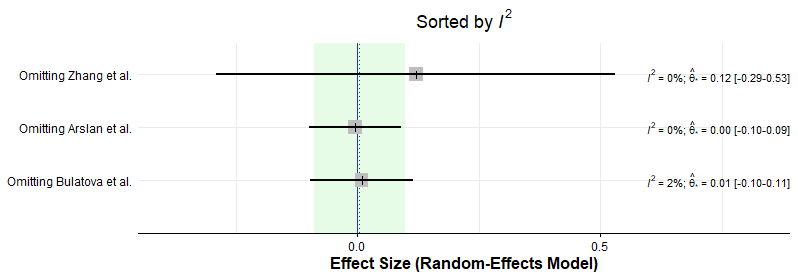


**Supplementary Fig. S14** Sensitivity analysis for waist circumference using the leave-one-out model.


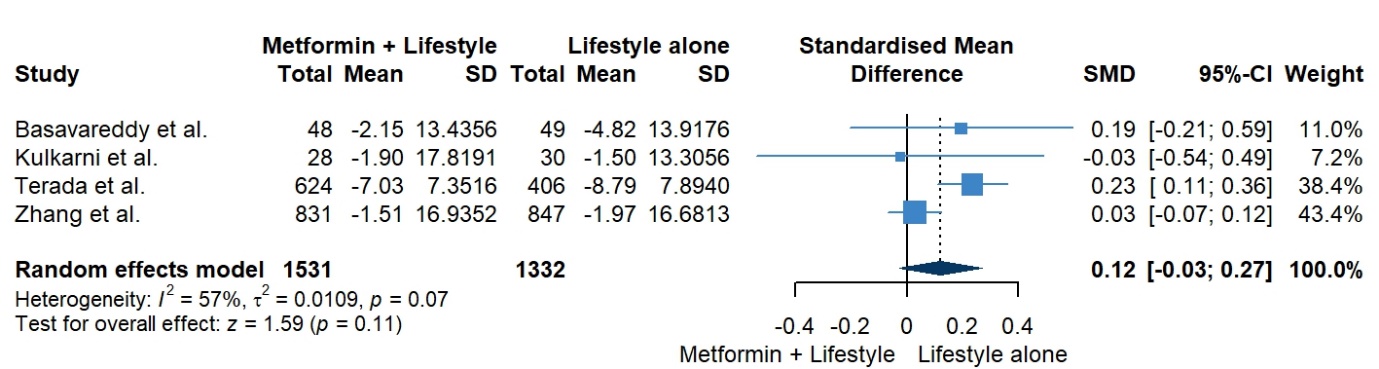


**Supplementary Fig. S15** Pooled studies for body weight.


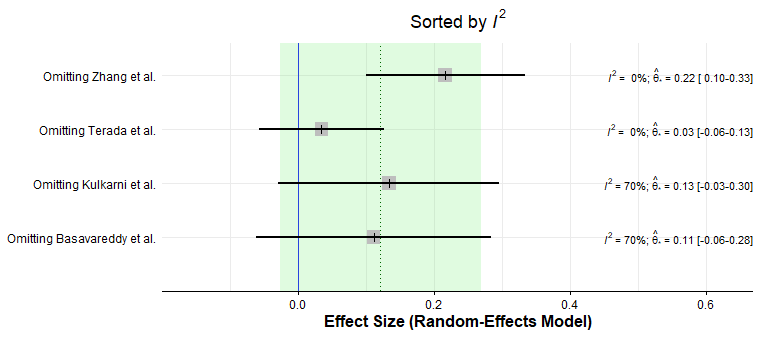


**Supplementary Fig. S16** Sensitivity analysis for body weight using the leave-one-out model.


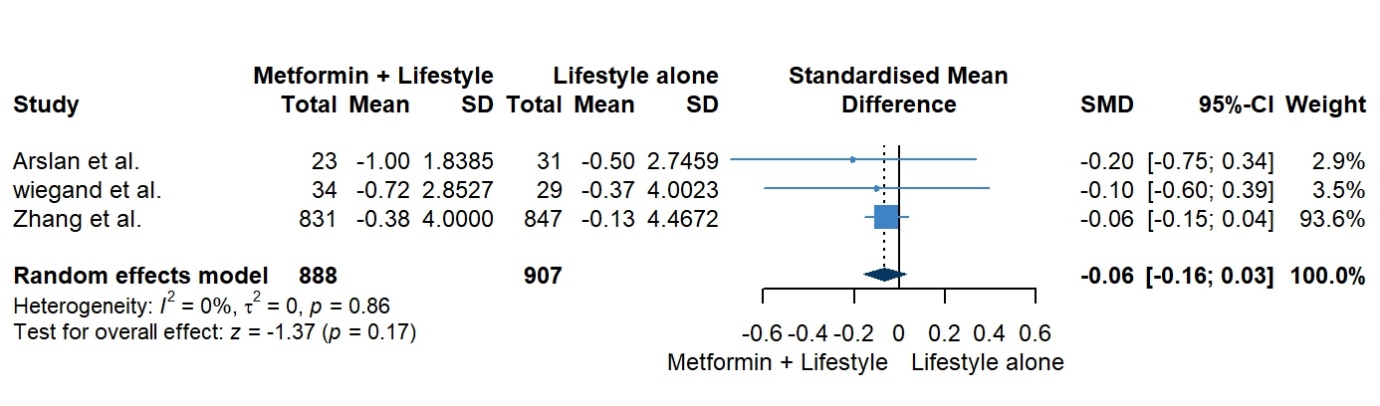


**Supplementary Fig. S17** Pooled studies for HOMA-IR.


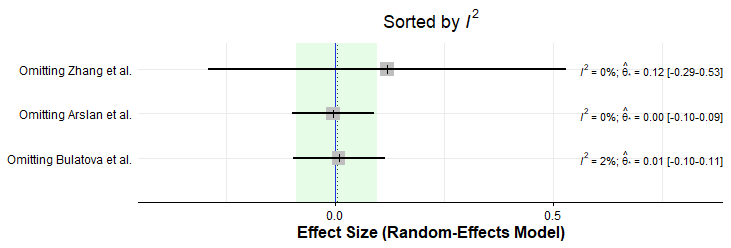


**Supplementary Fig. S18** Sensitivity analysis for HOMA-IR using the leave-one-out model.


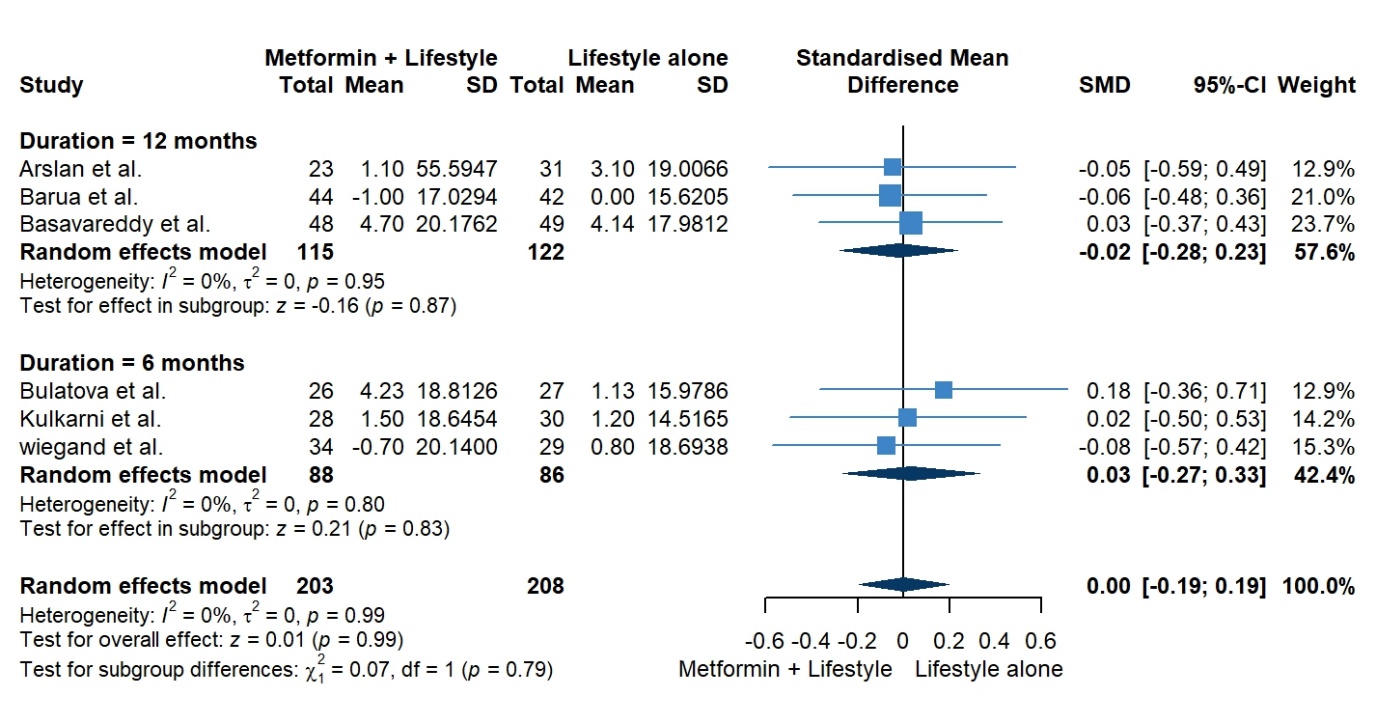


**Supplementary Fig. S19** Pooled studies for HDL at 6 and 12 months.


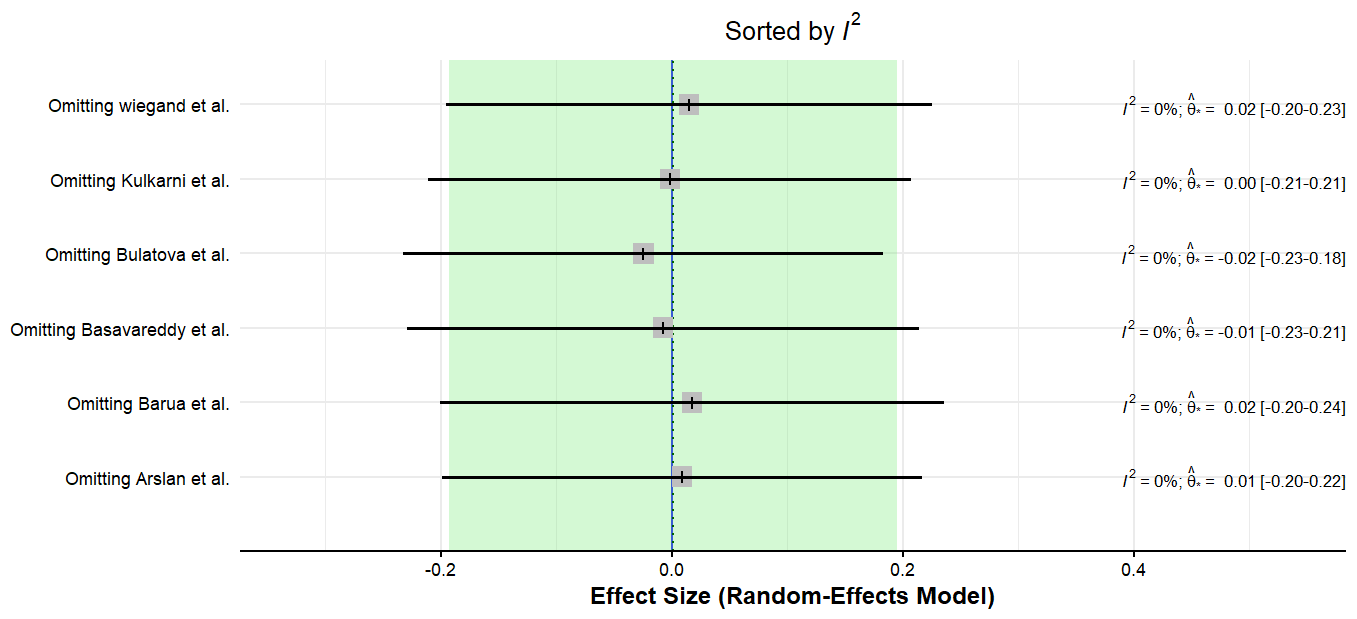


**Supplementary Fig. S20** Sensitivity analysis for HDL using the leave-one-out model.


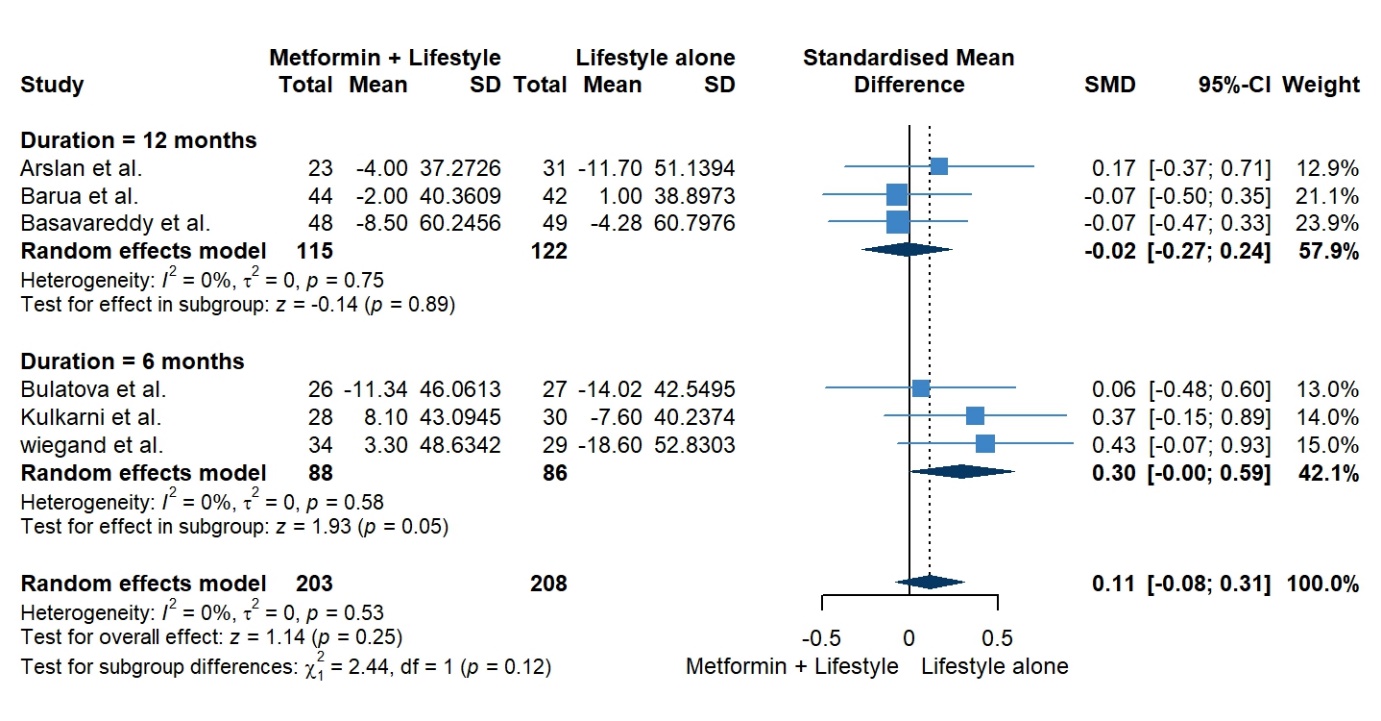


**Supplementary Fig. S21** Pooled studies for LDL at 6 and 12 months.


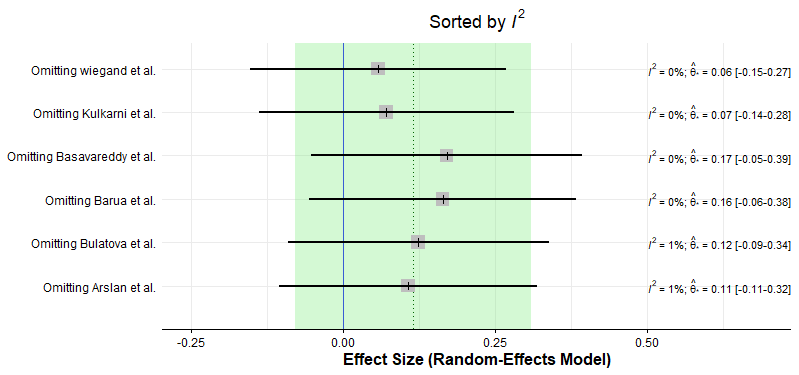


**Supplementary Fig. S22** Sensitivity analysis for LDL using the leave-one-out model.


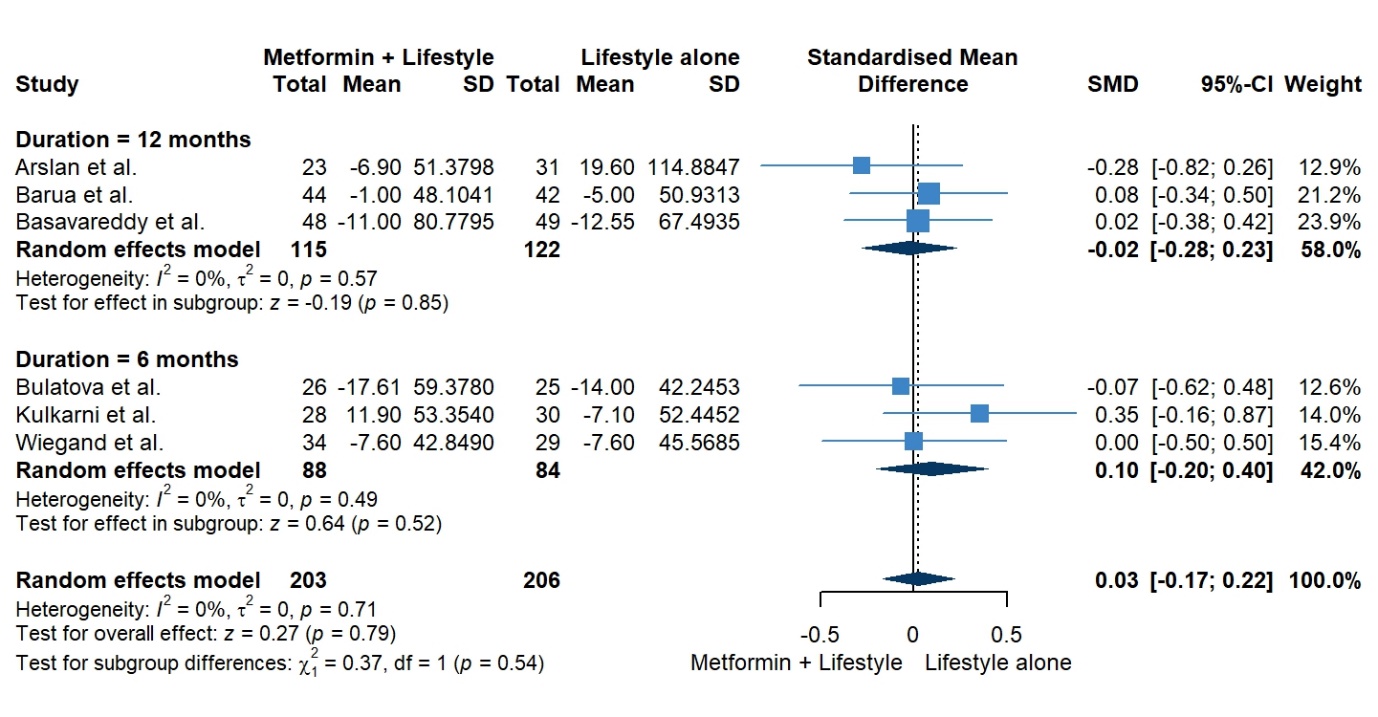


**Supplementary Fig. S23** Pooled studies for total cholesterol at 6 and 12 months.


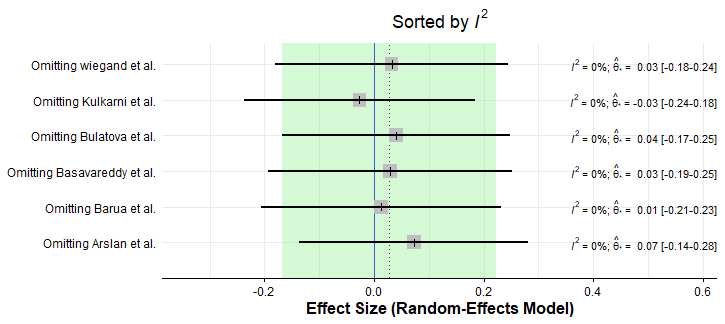


**Supplementary Fig. S24** Sensitivity analysis for total cholesterol using the leave-one-out model.


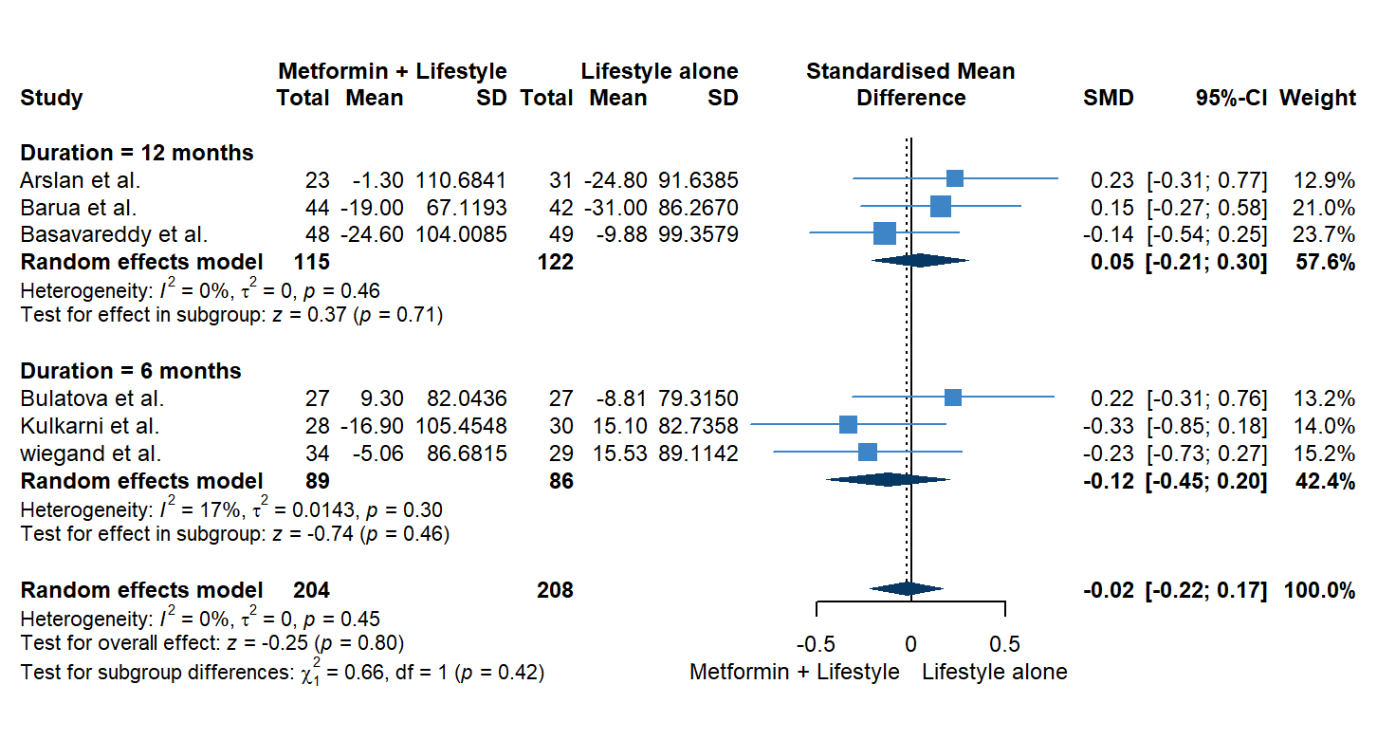


**Supplementary Fig. S25** Pooled studies for triglycerides at 6 and 12 months.

**Correspondence**

Basma Ehab Amer, Faculty of Medicine, Benha University, Benha, Egypt

Email: [basma.ehab15@gmail.com](mailto:basma.ehab15@gmail.com); [basma170140@fmed.bu.edu.eg](mailto:basma170140@fmed.bu.edu.eg)

ORCID: 0000-0001-7787-5508

**References**

1. Arslan MS, Tutal E, Sahin M, Karakose M, Ucan B, Ozturk G, Cakal E, Biyikli Gencturk Z, Ozbek M, Delibasi T (2017) Effect of lifestyle interventions with or without metformin therapy on serum levels of osteoprotegerin and receptor activator of nuclear factor kappa B ligand in patients with prediabetes. Endocrine 55:410–415. https://doi.org/10.1007/s12020-016-1121-4

2. Barua M, Pathan F, Nabi MU, Kabir M (2019) Assessment of clinical and biochemical profile of prediabetic subject in Bangladesh, attending in BIRDEM and results of intervention by lifestyle modification, metformin, and DPP4 inhibitor. Diabetes Metab Syndr Clin Res Rev 13:1603–1608. https://doi.org/10.1016/j.dsx.2019.03.019

3. Basavareddy A, Sarala N, Nanjappa V, Eshwarappa S (2022) A study of lifestyle modifications with and without metformin in prediabetic subjects. J Diabetol 13:277. https://doi.org/10.4103/jod.jod_40_22

4. Bulatova N, Kasabri V, Qotineh A, AL-Athami T, Yousef AM, AbuRuz S, Momani M, Zayed A (2018) Effect of metformin combined with lifestyle modification versus lifestyle modification alone on proinflammatory-oxidative status in drug-naïve pre-diabetic and diabetic patients: A randomized controlled study. Diabetes Metab Syndr Clin Res Rev 12:257–267. https://doi.org/10.1016/j.dsx.2017.11.003

5. Iqbal Hydrie MZ, Basit A, Shera AS, Hussain A (2012) Effect of intervention in subjects with high risk of diabetes mellitus in Pakistan. J Nutr Metab 2012:5–9. https://doi.org/10.1155/2012/867604

6. Kulkarni S, Xavier D, George D, Umesh S , Fathima S BG (2018) Effect of intensive lifestyle modification & metformin on cardiovascular risk in prediabetes: A pilot randomized control trial Shruthi. 148:705–712. https://doi.org/10.4103/ijmr.IJMR_1201_17

7. Love-Osborne K, Sheeder J, Zeitler P (2008) Addition of Metformin To a Lifestyle Modification. Heal (San Fr 152:817–822. https://doi.org/10.1016/j.jpeds.2008.01.018.ADDITION

8. Malin SK, Gerber R, Chipkin SR, Braun B (2012) Independent and combined effects of exercise training and metformin on insulin sensitivity in individuals with prediabetes. Diabetes Care 35:131–136. https://doi.org/10.2337/dc11-0925

9. Ramachandran A, Snehalatha C, Mary S, Mukesh B, Bhaskar AD, Vijay V (2006) The Indian Diabetes Prevention Programme shows that lifestyle modification and metformin prevent type 2 diabetes in Asian Indian subjects with impaired glucose tolerance (IDPP-1). Diabetologia 49:289–297. https://doi.org/10.1007/s00125-005-0097-z

10. Viskochil R, Malin SK, Blankenship JM, Braun B (2017) Exercise training and metformin, but not exercise training alone, decreases insulin production and increases insulin clearance in adults with prediabetes. J Appl Physiol 123:243–248. https://doi.org/10.1152/japplphysiol.00790.2016

11. Wiegand S, L’Allemand D, Hübel H, Krude H, Bürmann M, Martus P, Grüters A, Holl RW (2010) Metformin and placebo therapy both improve weight management and fasting insulin in obese insulin-resistant adolescents: A prospective, placebo-controlled, randomized study. Eur J Endocrinol 163:585–592. https://doi.org/10.1530/EJE-10-0570

12. Zhang L, Zhang Y, Shen S, Wang X, Dong L, Li Q, Ren W, Li Y, Bai J, Gong Q, Kuang H, Qi L, Lu Q, Cheng W, Liu Y, Yan S, Wu D, Fang H, Hou F, Wang Y, Yang Z, Lian X, Du J, Sun N, Ji L, Li G, Tu P, Wang S, Yin X, Chen W, He Q, Shan Z, Wang X, Liang J, Liu C, Xie Y, Duan B, Wang W, Gao Z, Li L, Jiang L, Han H, Chen X, Sun L, Feng B, Cao L, Wang X, Zhang T, Liu X (2023) Safety and effectiveness of metformin plus lifestyle intervention compared with lifestyle intervention alone in preventing progression to diabetes in a Chinese population with impaired glucose regulation: a multicentre, open-label, randomised controlled t. Lancet Diabetes Endocrinol 11:567–577. https://doi.org/10.1016/S2213-8587(23)00132-8
