# Supplementary material for: Metformin plus lifestyle interventions versus lifestyle interventions alone for the delay or prevention of type 2 diabetes in individuals with prediabetes: a meta-analysis of randomized controlled trials
Source: Diabetol Metab Syndr. 2024 Nov 14;16:273. doi: 10.1186/s13098-024-01504-8 (PMC11562588; doi:10.1186/s13098-024-01504-8)
Supplement: Supplementary file 2 — Supplementary Material 2 [file 13098_2024_1504_MOESM2_ESM.pptx]

## Slide 1
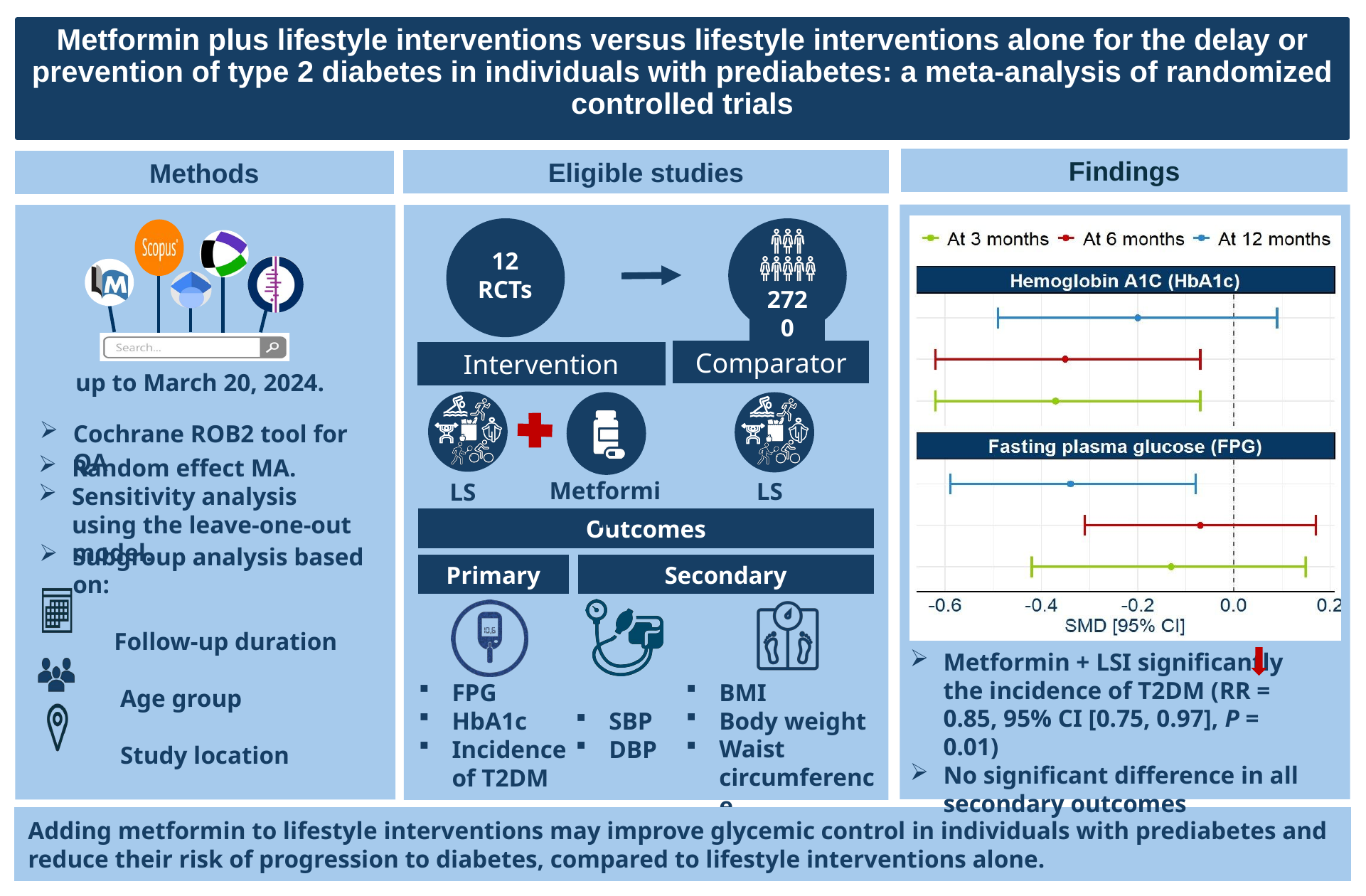

Metformin plus lifestyle interventions versus lifestyle interventions alone for the delay or prevention of type 2 diabetes in individuals with prediabetes: a meta-analysis of randomized controlled trials
Findings
Eligible studies
Methods
12 RCTs
2720
Comparator
Intervention
up to March 20, 2024.
Cochrane ROB2 tool for QA
Random effect MA.
Sensitivity analysis using the leave-one-out model.
Metformin
LSI
LSI
Outcomes
Subgroup analysis based on:
 Follow-up duration
 Age group
 Study location
Primary
Secondary
Metformin + LSI significantly the incidence of T2DM (RR = 0.85, 95% CI [0.75, 0.97], P = 0.01)
No significant difference in all secondary outcomes
BMI
Body weight
Waist circumference
FPG
HbA1c
Incidence of T2DM
SBP
DBP
Adding metformin to lifestyle interventions may improve glycemic control in individuals with prediabetes and reduce their risk of progression to diabetes, compared to lifestyle interventions alone.
